# The unplumatellid *Plumatella fruticosa* found its home: *Hirosella* gen. nov. morphological arguments for the systematic placement of a freshwater bryozoan

**DOI:** 10.1002/jmor.21620

**Published:** 2023-08-04

**Authors:** Julian Bibermair, Thomas Schwaha

**Affiliations:** ^1^ Department of Evolutionary Biology University of Vienna Vienna Austria

**Keywords:** 3D reconstruction, Bryozoa, *Hirosella fruticosa*, lophophorata, myoanatomy, new genus, Phylactolaemata

## Abstract

Bryozoans are colonial, suspension‐feeding lophotrochozoans. The phylum consists of the large group of chiefly marine Myolaemata and the exclusively limnic Phylactolaemata. Each colony consists of individual zooids that comprise the protective cystid and the retractable polypide. Phylactolaemates are a small group of approximately 90 species in 6 families. They feature a body wall, that can either be gelatinous, as in the families Stephanellidae, Lophopodidae, Cristatellidae and Pectinatellidae, or encrusted, as in Plumatellidae and Fredericellidae. Morphological investigations of the most specious plumatellids are rare and focus on few species. *Plumatella fruticosa* is of particular interest in this regard, as it shows a mosaic of plumatellid and fredericellids characters. The most recent phylogeny clusters *P. fruticosa* with cristatellids and pectinatellids as sister groups to fredericellids. Hence, there is considerable doubt, whether *P. fruticosa* is truly a plumatellid. Therefore, this study aims to reinvestigate the morphology of *P. fruticosa* with confocal microscopy and section‐based three‐dimensional reconstruction. The new data show that *P. fruticosa* has numerous conspicuous stumps from fragmented proliferation buds, which are otherwise only known from fredericellids. Like fredericellids, *P. fruticosa* grows erect, but in contrast, has a horseshoe‐shaped lophophore and floatoblasts. Besides the proportions of the lophophore, the tentacle sheath and digestive tract resemble a fredericellid‐like situation. Myoanatomical details like the pronounced longitudinal muscles of the vestibular wall and tentacle sheath differ from plumatellids and favour the recently proposed scenario, which places *P. fruticosa* next to Pectinatellidae and Cristatellidae. In addition, the intertentacular membrane of *P. fruticosa* shows structural similarity to cristatellids as it is attached to the tentacles via lamellae. Taking all aspects into account, we erect a new family: Hirosellidae fam. nov. including the new genus *Hirosella* gen. nov.

## Graphical abstract

The freshwater bryozoan *Plumatella fruticosa* has been ambiguously discussed, as it shows characters of several phylactolaemate clades. Besides the serrated body wall our morphological analysis found myoanatomical details unique for this species. Proportions of the digestive tract and tentacle sheath, the musculature of the latter, and lamellae of the intertentacular membrane allow to argue in favour of a recently proposed phylogeny. Thus, we introduce a new genus and its separate family: *Hirosella* gen. nov. with the type species *Hirosella fruticosa*.

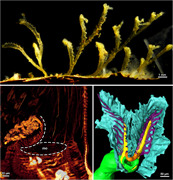

## 1 INTRODUCTION

Phylactolaemates constitute a small group within Bryozoa, a lophotrochozoan phylum of colonial, sessile and suspension‐feeding coelomates. The whole phylum includes over 6000 extant species divided into several taxa: the gymnolaemates and the stenolaemates (cyclostomes) can be summarised as predominantly marine myolaemates, which mostly feature a mineralised body wall (Bock & Gordon, 2013; Schwaha et al., 2020). The phylactolaemates represent the sister group to the Myolaemata (Saadi et al., 2022; Schwaha et al., 2020; Taylor & Waeschenbach, 2015; Waeschenbach et al., 2012). Phylactolaemates are the only clade exclusively found in freshwater habitats and lack a calcified body wall (Massard & Geimer, 2008; Mukai et al., 1997; Schwaha, 2020a; Wood, 2015).

Individual zooids of a colony comprise a cystid and a polypide. The former constitutes the body wall with a peritoneal and epidermal layer. In phylactolaemates, an orthogonal grid of body wall muscles is embedded in the extracellular matrix of both layers (Bibermair et al., 2022; Hyatt, 1866; Marcus, 1934; Schwaha, 2020b; Schwaha & Wanninger, 2012). In addition, the cystid produces a cuticle (ectocyst) that can either be gelatinous or encrusted in phylactolaemates. The retractable polypide mainly consists of a lophophore with ciliated tentacles, a U‐shaped digestive tract and a central nervous system located between the descending (pharynx) and ascending (intestine) arms of the gut. Phylactolaemates show specific characteristics in their gross morphology. Their lophophore is horseshoe‐shaped, with two lophophoral arms extending at the ‘back’ (anal) side (Mukai et al., 1997; Wood, 2015; Wood & Okamura, 2005). In the proximal area of the lophophoral base, a thin duplicature connects the proximal side of neighbouring tentacles. This membrane is called the intertentacular membrane and is apomorphic for phylactolaemates (Braem, 1890; Gawin et al., 2017; Schwaha & Hirose, 2020; Schwaha et al., 2020). A flap‐ or dome‐shaped epistome is also situated at the lophophoral base and arches over the mouth opening in the oral direction of the polypide. This structure is also apomorphic for phylactolaemates (Wood, 1983).

All bryozoan colonies grow by asexual budding. In phylactolaemates, the development of new buds is with one exception restricted to the oral side. They also produce internal, encapsulated buds as so‐called statoblasts that serve for overwintering and dispersal, and which are crucial for species identification (Wood, 1983; Wood et al., 2006; Wood & Okamura, 2005).

Morphologically, seven families are recognised among phylactolaemates, although one (Tapajoselidae) is solely based on statoblast morphology, without any living specimens encountered so far (Wood & Okamura, 2017). Consequently, six families are supported by molecular data as well (Hartikainen et al., 2013; Hirose et al., 2008; Massard & Geimer, 2008; Saadi et al., 2022; Waeschenbach et al., 2012). Four families form gelatinous ectocysts: Stephanellidae, Lophopodidae, Cristatellidae and Pectinatellidae; the latter three also form clustered colonies.

Colony morphology, the number of tentacles and the shape of the lophophore are helpful characters when assigning specimens to families. The families Fredericellidae and Plumatellidae include mostly a chitinous/encrusted ectocyst and show a serial arrangement of zooids. Fredericellids comprise the genera *Fredericella* and *Internectella* and are the only family with a circular lophophore, which is a secondary feature (Gruhl & Bartolomaeus, 2008; Gruncharova, 1971; Marcus, 1926). In addition, fredericellids develop a unique form of statoblasts called piptoblasts (Wood, 2015; Wood & Backus, 1992; Wood & Okamura, 2005). In contrast to the floatoblasts that are found in most other phylactolaemates, piptoblasts lack a gas‐filled annulus. The presence of piptoblasts and the circular lophophore makes them easily distinguishable from plumatellids. The latter are the most speciose group of phylactolaemates and are nowadays considered as late‐branching within the tree. Specimens with an encrusted cystid, a horseshoe‐shaped lophophore and ideally also free floatoblasts and sessile sessoblasts are identified as plumatellids (Wood & Okamura, 2005; Wood et al., 2006).

*Plumatella fruticosa* displays a mosaic of fredericellid and plumatellid characters: (1) Aforementioned characters apply to *P. fruticosa*. (2) Colony morphology is reminiscent of fredericellids in terms of erect zooids with slender cystids. (3) Sexually produced larvae of *P. fruticosa* have one polypide, like fredericellids (Allman, 1856; Braem, 1908) and unlike plumatellids, which have two (Bibermair et al., 2021; Braem, 1897). (4) The intertentacular membrane shows similarity to *Cristatella mucedo* (Braem, 1890). Although not resolving its phylogenetic position, first molecular analyses also showed that *P. fruticosa* was not grouped with the remaining Plumatellidae (Hartikainen et al., 2013). Recently, a transcriptome‐based phylogeny confirmed that *P. fruticosa* is not a plumatellid and is sister group to *Cristatella* and *Pectinatella*, a clade referred to as pectinatella–cristatella–plumatella (PCP)‐clade (Saadi et al., 2022). Together with Fredericellidae the PCP‐clade is a sister to plumatellids, which confirms *P. fruticosa* is more closely associated with fredericellids. Our understanding of whether there are morphological characters favouring the PCP‐clade and the support of the sister‐group relationship to fredericellids is hindered by the absence of modern analyses. This study aims to provide morphological evidence that supports the PCP‐clade and a close relationship of *P. fruticosa* to fredericellids. For that purpose, confocal microscopy and histology combined with three‐dimensional (3D) reconstruction were used to study the myoanatomy and general morphology of *P. fruticosa*. In addition, comparative data was also gathered from Fredericellidae.

## 2 MATERIALS AND METHODS

### 2.1 Specimen collection

Colonies of *P. fruticosa* Allmann, 1844 (=*Hirosella* gen. nov. *fruticosa*, which will be subsequently used throughout the manuscript, see Section 3.6) were sampled in the Hirzmann barrier lake (47°00′33.2″N 15°03′29.1″E) in August 2020 and a pond of Klosterneuburg area (48°19′26.3″N 16°19′23.0″E) in July 2022, both in Austria. Before fixation, samples were imaged and filmed using a Nikon Ds‐Ri2 camera mounted on a Nikon SMZ 25 microscope (Nikon). Some samples were relaxed using cocaine hydrochloride. Colonies were fixed in 4% paraformaldehyde (PFA) in 0.1 mol L^−1^ phosphate buffer (PB, pH 7.3, aqueous solution with 0.075 mol l^−1^ Sodium Phosphate Dibasic Heptahydrate and 0.025 mol l^−1^Sodium Phosphate Monobasic Monohydrate) for approximately 1 h and rinsed several times in the same buffer. Samples were stored in 0.1 mol L^−1^ PB including ∼0.1% NaN_3_ until further preparation. In addition, *Fredericella sultana* (Blumenbach, 1799) was sampled in Austria and Kanchanaburi, Thailand, *Internectella bulgarica* in Kanchanaburi, Thailand, in 2009. For comparison, ethanol‐fixed specimens of *Gelatinella toanensis*, *Plumatella* cf. *philippinensis* from the Zoological Museum Hamburg were included in this study. *Plumatella fungosa* was sampled in Austria, in areas around Vienna. *Plumatella casmiana* and *Plumatella bombayensis* were sampled from the pond of the Faculty of Fisheries at Kasetsart University, Bangkok, Thailand, in 2009 and 2020. *Rumarcanella vorstmani* was sampled in Sakhon Nakon, Thailand, in 2020. *Hyalinella punctata*, *Pectinatella magnifica*, and *Cristatella mucedo* were sampled in Austria from local ponds in and around Vienna from 2019 to 2022.

### 2.2 Confocal microscopy

For immunocytochemistry and confocal laser scanning microscopy (CLSM) colony pieces were dissected into individual zooids and freed from the ectocyst, occasionally the entire cystid. To improve permeability, the specimens were treated with 0.1 mol L^−1^ PB including 2% Triton‐X 100 and 2% dimethylsulphoxide (PBT) for 24 h. For Factin staining, Alexa flour 488 phalloidin (Cat# A12379; Thermo Fisher Scientific) was applied at a dilution 1:40 and nuclear‐counterstaining was done using 4′,6‐diamidino‐2‐phenylindole (Invitrogen) at a dilution approximately 1:300. After staining, the specimens were rinsed several times in 0.1 mol L^−1^ PB and mounted on object slides in Flouromount G (Southern Biotech). Scans were carried out on a Leica TCS SP5 II CLSM (Leica Microsystems). Image processing and analysis were done using FIJI (Schindelin et al., 2012) and Amira software (v. 2022; Thermo Fischer Scientific). Individual muscles were segmented using the segmentation editor of Amira, volume renderings were produced using the volren and volume rendering module in combination with several orthoslices. Snapshots were exported to be further processed using Adobe Photoshop.

### 2.3 Histology and 3D reconstruction

PFA‐fixed samples were postfixed in 1% aqueous osmium tetroxide for 1 h and rinsed several times in purified water. After osmification, samples were dehydrated in acidified 2,2‐dimethoxypropane for 30 min and infiltrated with low‐viscosity resin (Agar Scientific) overnight with pure acetone as intermedium. Specimens were placed in silicone moulds and polymerised at 60°C overnight.

Ribbons of 1‐µm‐thick serial sections were produced using a Histo Jumbo knife (Diatome) on a Leica UC6 ultramicrotome (Leica Microsystems) in accordance with established protocols (Ruthensteiner, 2008). Sections were stained with 1% toluidine blue (40 s, 60°C). Image stacks were prepared with a Nikon Ds‐Ri2 camera mounted on a Nikon Ni‐U compound microscope (Nikon).

The image stack was transferred into 8‐bit greyscale and checked for images unsuitable for reconstruction using the stack sorter tool in FIJI (Schindelin et al., 2012). The prepared image stack was registered into the Amira software, where it was semiautomatically aligned and resampled via the AlignSlices module. Organs and structures of interest were manually segmented in the segmentation editor of Amira. If applicable, structures such as the digestive tract and the retractor muscles were presegmented in Amira and the resulting label files as well as the corresponding alignments uploaded into the Biomedisa platform (Lösel et al., 2020) for semiautomatic segmentation. Final labels were further processed in Amira. A surface of all segmented materials was created using the GenerateSurface module. The surfaces were optimised via several alternating triangle reduction and smoothing steps. The final 3D reconstructions were visualised with several SurfaceView modules, partly in combination with volume renderings. Animations of some reconstructions were created via the animation editor of Amira. Calibrated snapshots were exported from Amira and also further processed using Fiji and Photoshop.

## 3 RESULTS

### 3.1 Colony morphology

**Figure 1:**
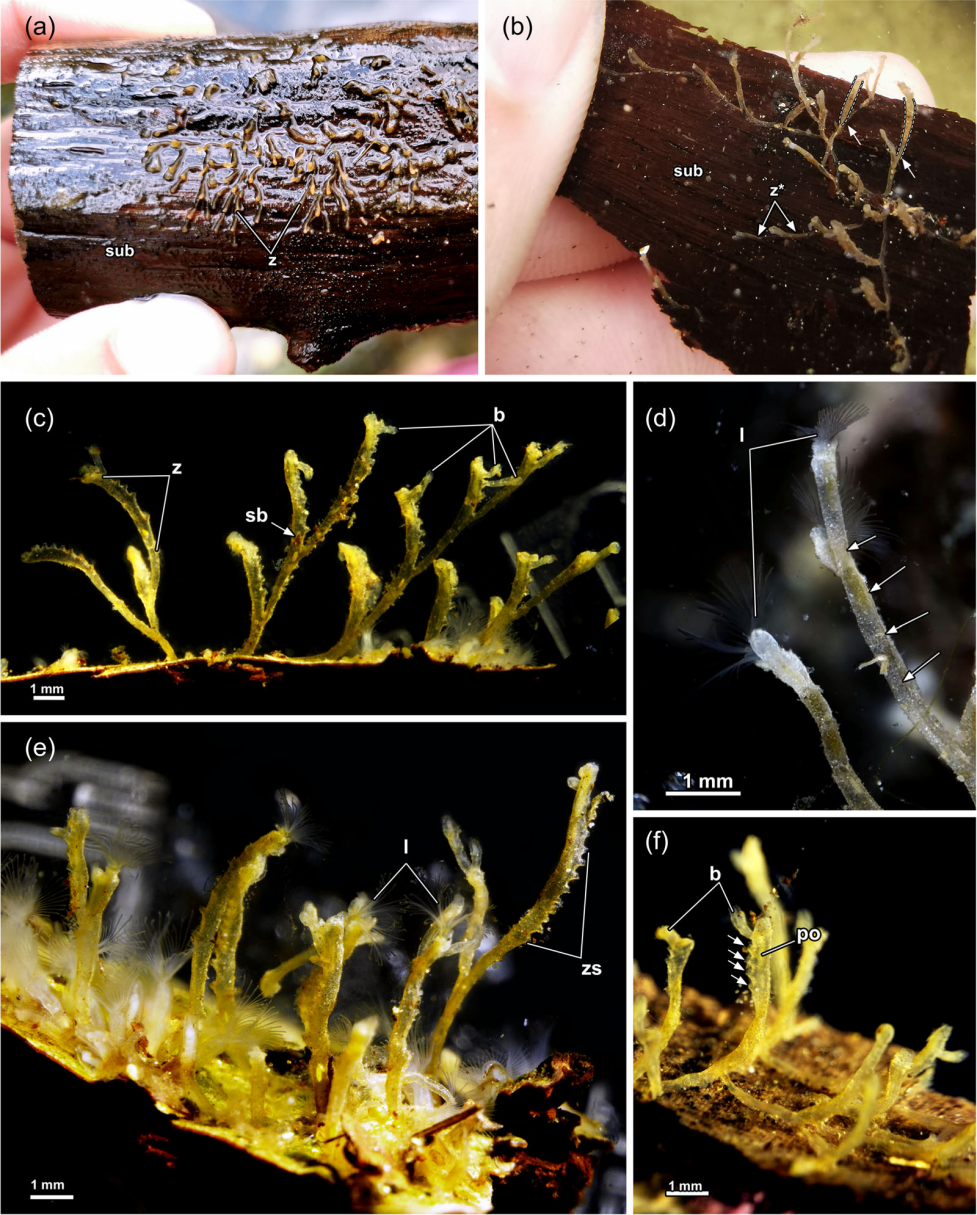
Colonies of *Hirosella fruticosa*. (a, b) Dense‐packed colonies of *H. fruticosa* growing on dead wood. The erect colonies collapse when above the water (a), in submerged condition (b) the zooids are barely attached to the substrate (sub) and have a cylindrical cystid with the distal end wider than its base (arrows). Young zooids become erect as they grow. (c–f) The colony features erect main stems of which each includes several buds, that ultimately produce buds on their own (c). Cystids have a keel on younger, less encrusted zooids (d, arrows). Cystid consistently shows a serrated appearance (e). Referred appearance results from proliferating buds which break off and leave behind up to 15 successively arranged stumps (e, f, arrows). Fragmentation starts in young zooids, with the youngest bud located most distal in the zooid (f). b, bud; l, lophophores; po, polypide; sb, statoblast; z*, creeping zooid; zs, serrated zooid.

**Figure 2:**
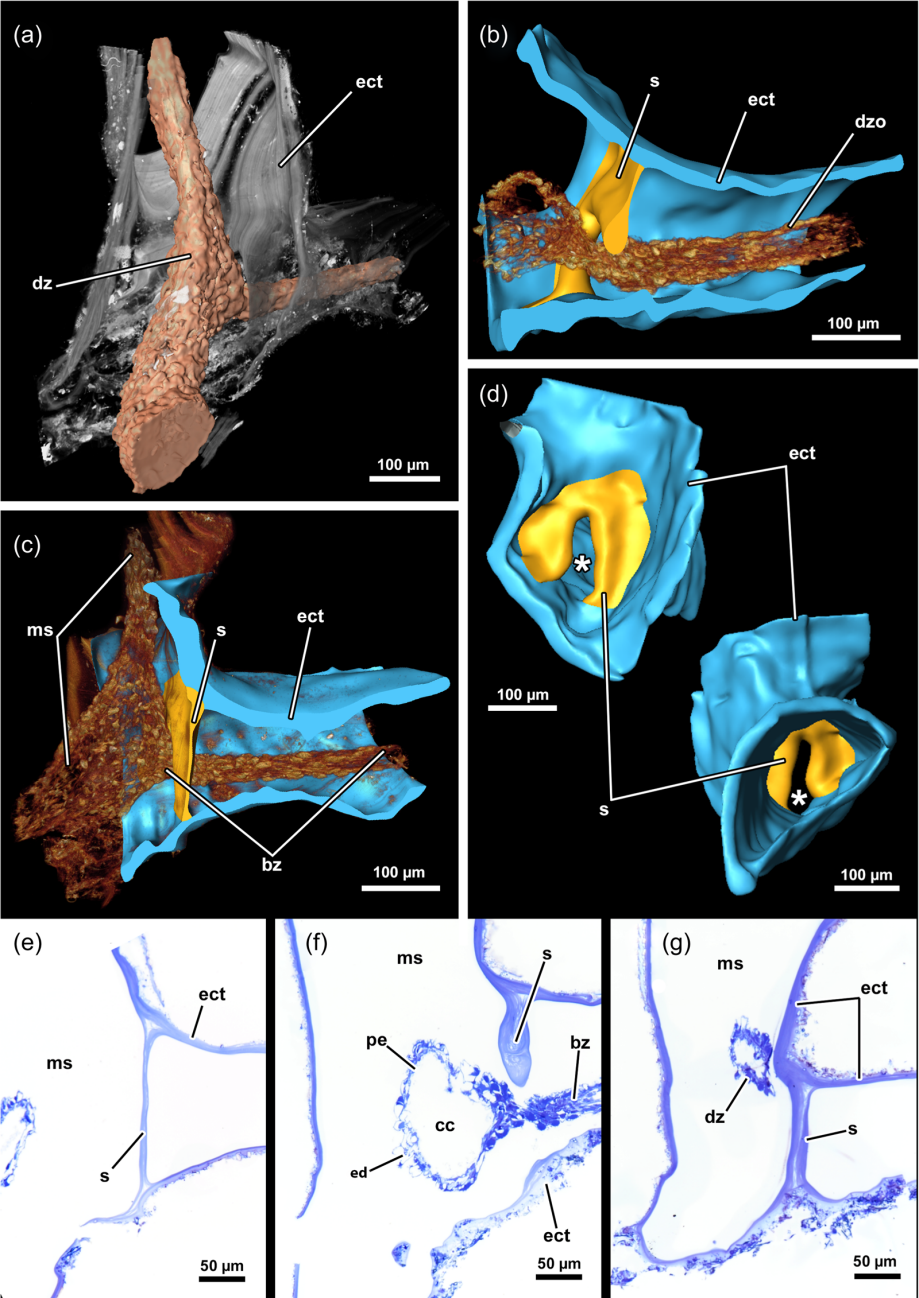
Three‐dimensional (3D) reconstruction and histology of the incomplete, interzooidal septum of *Hirosella fruticosa*. (a) Volume rendering of a colony piece with degenerated, shrunken zoid. (b–d) 3D reconstruction of the septum and its interzooidal connection (b). This septum is thickest in the centre (b) and becomes thinner towards its periphery (c). The septum is incomplete and has a gap in the centre (d). In cross‐section, the shape of the cystid is triangular. (e–g) Histological serial sections show varying thickness of the septum, that is thinnest at its margin (e), thickest at the central gap (f) and comparatively thick at the periphery of the gap (g). bz, branching zooid; cc, coelomic cavity; dz, degenerated zooid; ect, ectocyst; ed, epidermis; ms, main stem.

**Figure 3:**
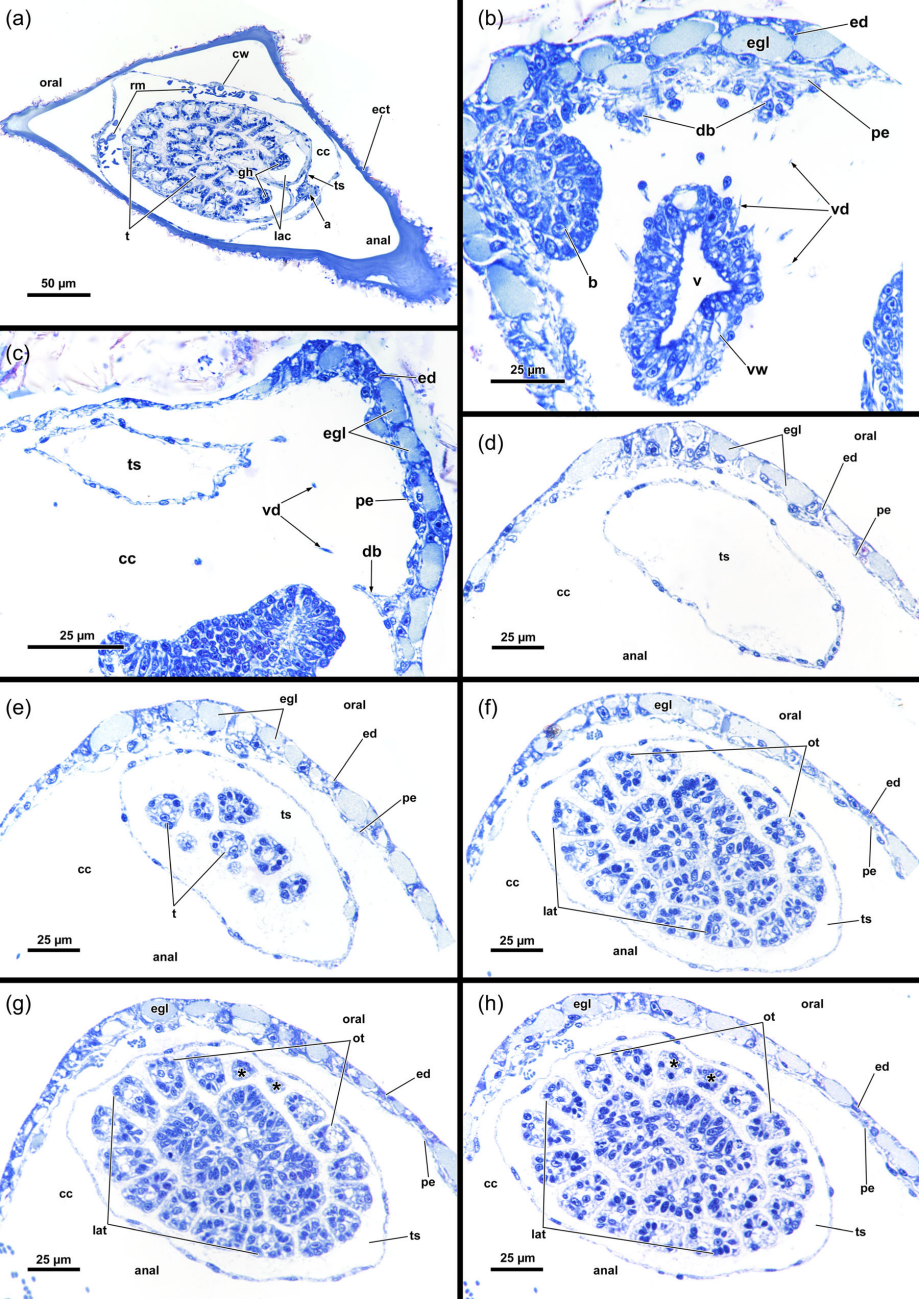
Histological sections of the distal area of *Hirosella fruticosa*. (a) Triangular cross‐section of the ectocyst. On the oral side retractor muscles (rm) project from the body wall to the tentacle sheath (ts). (b) Vestibular wall (vw) is connected to the body wall via individual vestibulum dilatators (vd). Duplicature bands (db) project from the peritoneum of the body wall towards the vw. (c, d) Proximal of the vestibulum, the ts continues as thin, bilayered epithelium. In the vestibular region db project towards the ts (c). In contrast to the vestibular region of the ts, the bilayered epithelium is thin in the proximal region of the former (d). (e–h) Only the proximal half of the ts is occupied by tentacles. The tentacles of the lophophoral arms extend most (e) followed by lateral to oral tentacles (ot) (f). The most ot are frequently shorter than the rest (g, h, asterisks). The proximal region of the ts lacks db. a, anus; b, bud; cc, cystid coelom; cw, cystid wall; ed, epidermis; egl, epidermal gland cells; gh, ganglion horns; lac, lophophoral arm coelom; lat, lophophoral arm tentacles; p, peritoneum; t, tentacle; v, vestibulum.

The collected specimens were found to grow on dead wood submerged in stagnant water (Figure 1a,b). Several colonies were large and featured erect zooids with encrusted cystids (Figure 1c,e,f). Also, young colonies barely show any bud formation yet grew upright instead of creeping along the substrate (Figure 1d). Individual zooids are narrow at their base but tend to broaden towards the distal end (Figure 1b,c,e,f). Frequently, a keel is present on the oral side of the cystid wall. However, it is only marginally developed and best seen in young zooids before they become strongly encrusted (Figure 1d). In cross‐section, zooids are often triangular (Figures 2d and 3a). As the zooids grow, they often feature a serrated cystid on the oral side (Figure 1c,e,f). This results from the sequential formation of several buds that had eventually broken off from the main stem. Nevertheless, young buds that grow in a distal direction were present (Figure 1e,f). An incomplete septum where zooids branch off occurs consistently between zooids (Figure 2a–c,f). The ring‐like septum features a central gap or, more precisely, a narrow slit (Figure 2d) that maintains interconnectivity between zooids (Figure 2b,c,f). The incomplete septum is a cuticular fold of the ectocyst (Figure 2e,f). It is comparatively thin in the peripheral area (Figure 2e), but thicker towards the centre (Figure 2f,g).

### 3.2 Zooid morphology

**Figure 4:**
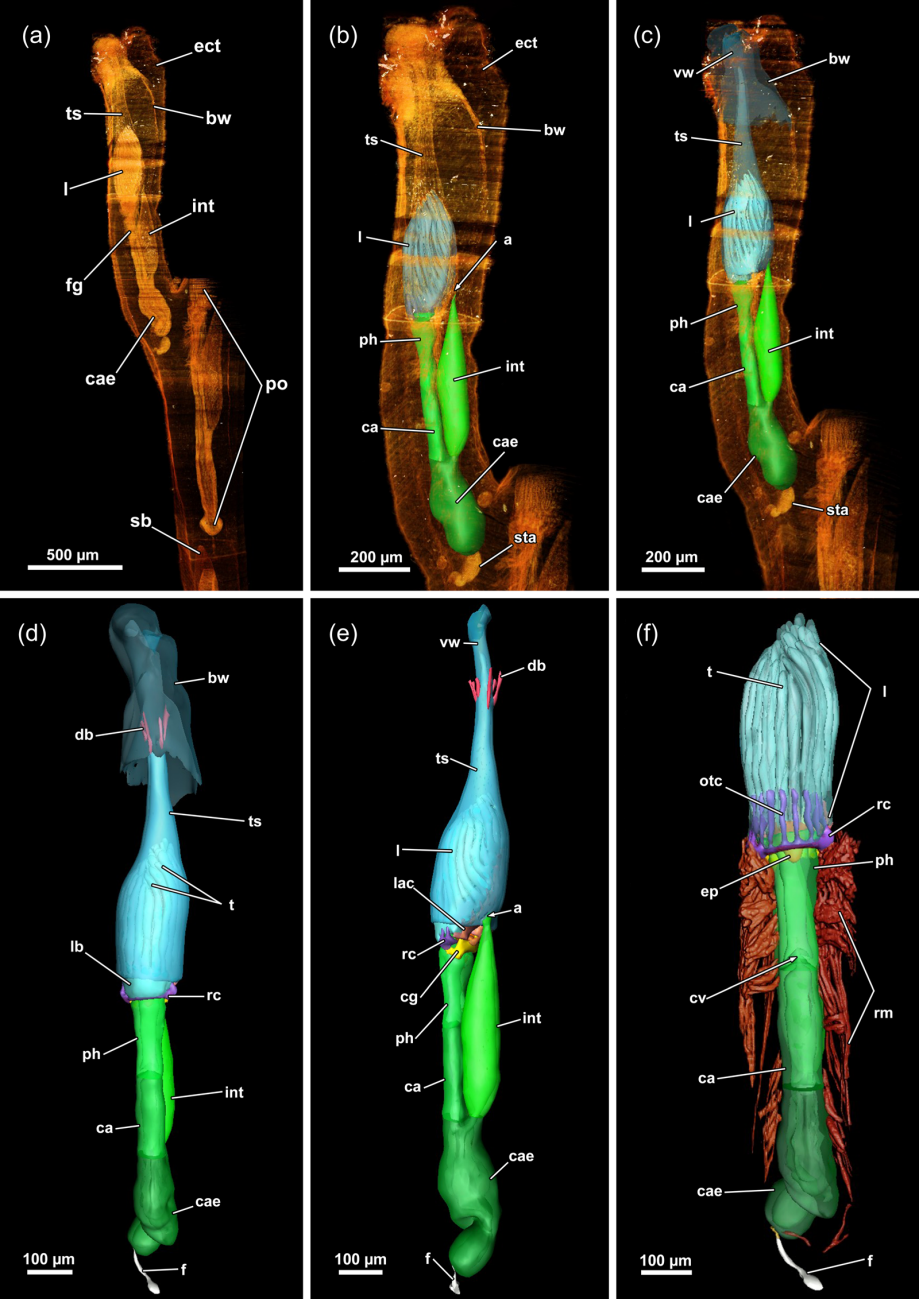
Three‐dimensional (3D) reconstruction of an individual polypide of *Hirosella fruticosa*. (a) The cystid is triangular in cross‐section with the tapered end located at the anal side. Polypides with the pronounced longitudinal axis are retracted into the cystid. (b, c) The digestive tract of the zooid is elongated, with the lophophore approximately half its length (b). When retracted, the lophophore occupies slightly more than half of the tentacle sheath (ts) (c). (d, e) Frontal (d) and lateral (e) view of an individual polypide. Duplicature bands (db) insert at the vestibular region of the ts (e). (f) Sets of prominent retractor muscles (rm) attach at various parts of the digestive tract and are most abundant in the distal region of the latter. An epistome is present as a beak‐like protrusion above the pharynx (ph). a, anus; bw, body wall; ca, cardia; cae, caecum; cg, cerebral ganglion; ect, ectocyst; ep, epistome; f, funiculus; fg, foregut; int, intestine; l, lophophore; lac, lophophoral arm coelom; otc, oral tentacle coelom; ph, pharynx; rc, ring canal; sb, statoblast; sta, statoblast anlage; t, tentacle; vw, vestibular wall.

**Figure 5:**
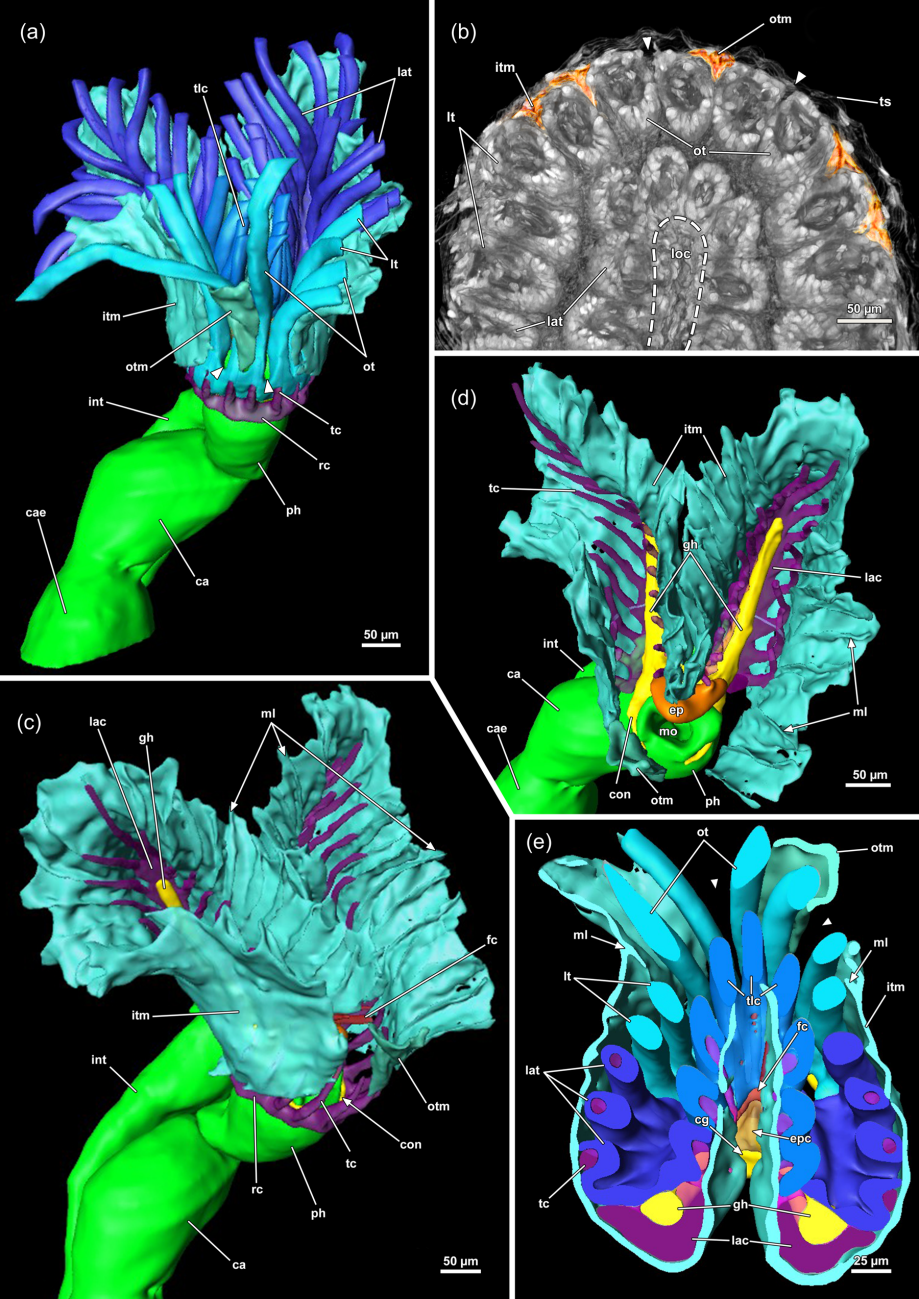
Protruded polypide of *Hirosella fruticosa*. (a) Frontal view showing the intertentacular membrane between the proximal tentacle area. Adjacent to the oral‐most tentacles the membrane has a gap (arrowheads). (b) Cross‐section of a retracted polypide with the intertentacular membrane highlighted between the oral tentacles (ot). A gap is present next to the oral‐most tentacles (arrowheads). (c, d) Oblique view of the lophophoral base without tentacles. The tentacle coelom (tc) ascends from the ring canal (rc) on the oral side (c), respectively, the lophophoral arm coelom (lac) (d) into the tentacles. Membranous lamellae are present on the inside of the intertentacular membrane. At the base of the lophophore, a circumoral nerve ring projects from the cerebral ganglion (cg) orally. On the anal side, two ganglionic horns (gh) extend into the lophophoral arms. (e) Cross‐section of the lophophoral base with lamellae attaching the intertentacular membrane to the tentacles. ca, cardia; cae, caecum; ep, epistome; fc, forked canal; gh, ganglionic horns; int, intestine; itm, intertentacular membrane; lat, lophophoral arm tentacles; loc, lophophoral concavity; lt lateral tentacles; ml, membranous lamella; mo, mouth; otc, oral tentacle coelom; otm, membrane between oral‐most tentacles; ph, pharynx; tlc, tentacles of the lophophoral concavity.

**Figure 6:**
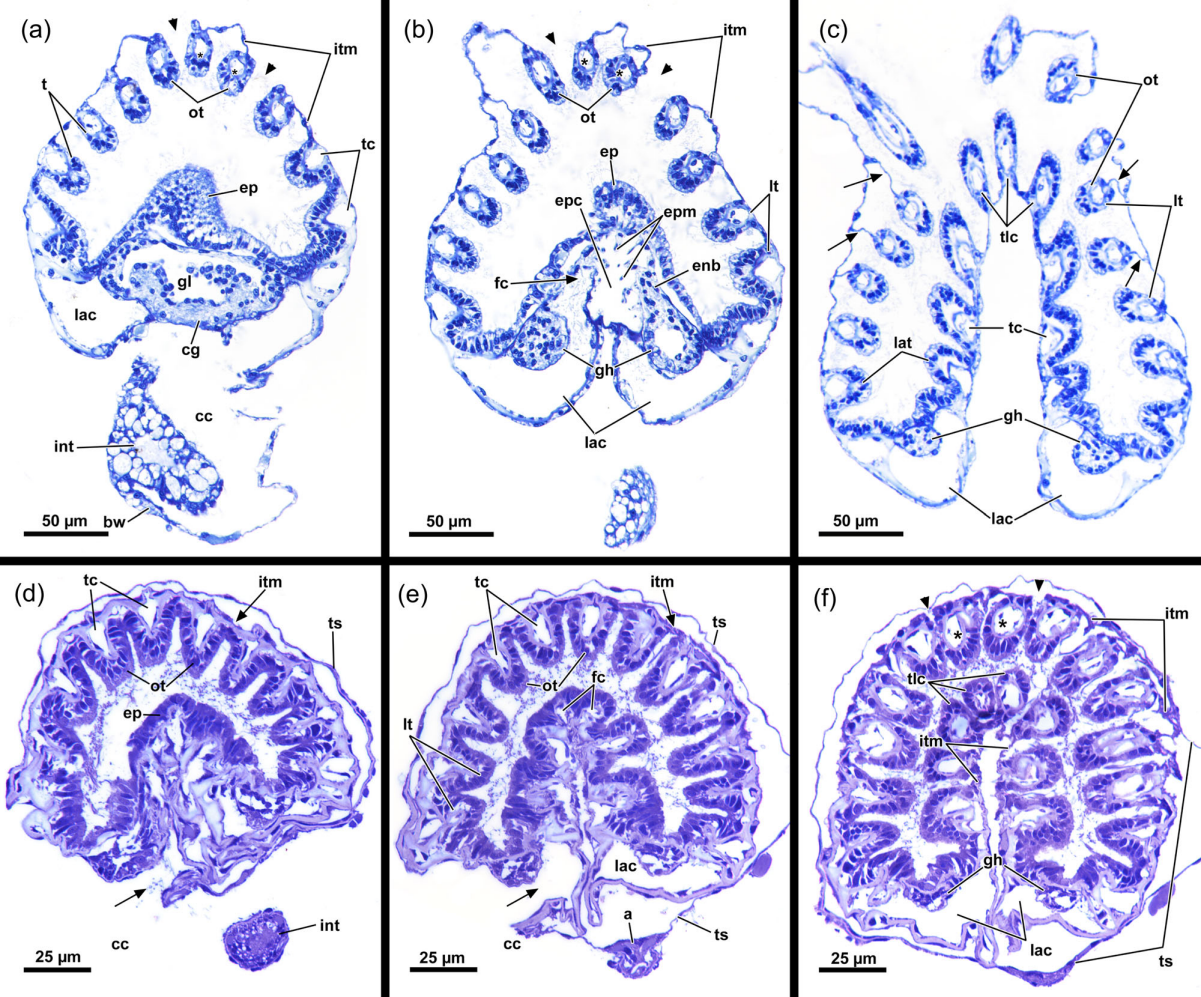
Histological cross‐sections of lophophoral base and lophophore of *Hirosella fruticosa* in protruded (a–c) and retracted condition (d–f). (a) Section through the proximal epistome (ep) area, cerebral ganglion (cg) and base of the lophophoral arms. The intertentacular membrane is present between the oral‐most tentacles (asterisks) and between the lateral tentacles. The membrane has a gap at the oral‐most tentacles (arrowheads). (b) Transversal ep muscles within the ep cavity. The intertentacular membrane is attached to the oral and lateral tentacles via a lamella. (c) More distal section showing a lamella projecting to individual tentacles. (d) Intertentacular membrane at the lophophoral base. The ep is present as thickened epithelium above the mouth opening. (e) Section through a more distal region of the lophophore shows the intertentacular membrane as a bilayered duplicature of the epidermis between the tentacles. (f) The intertentacular membrane spans between all tentacles and often has a peak pointing towards the space between the tentacles. a, anus; bw, body wall; cc, cystid coelom; enb, epistome neurite bundle; epc, epistome coelom; epm, epistome muscles; fc, forked canal; gh, ganglionic horns; gl, ganglion lumen; int, intestine; itm, intertentacular membrane; lac, lophophoral arm coelom; lat, lophophoral arm tentacles; lt, lateral tentacles; ot, oral tentacle; t, tentacle, tc, tentacle coelom; tlc, tentacles of the lophophoral concavity; ts, tentacle sheath.

In retracted conditions, the cystid enters the vestibular wall at the distal end of each zooid. Proximally it continues as a tentacle sheath (Figure 4a–e) and surrounds the retracted lophophore. The lophophore includes at least 40 tentacles in all sectioned specimens of *Hirosella fruticosa*. The intertentacular membrane is a duplicature of the epidermal layer and is spanned between the tentacles (Figures 5 and 6b,c,f) and covers approximately a third of the tentacle height. At the base, the membrane is broad, directly at the abfrontal lateral border of each tentacle (Figure 6d,e), whereas distally it attaches to individual tentacles on the medioabfrontal side via a lamella or peg (Figures 5d,e and 6a–c). A gap in the intertentacular membrane is present next to the oral‐most pair of tentacles (Figures 5a,b,f [arrows] and 6a–c,f [arrows]).

**Figure 7:**
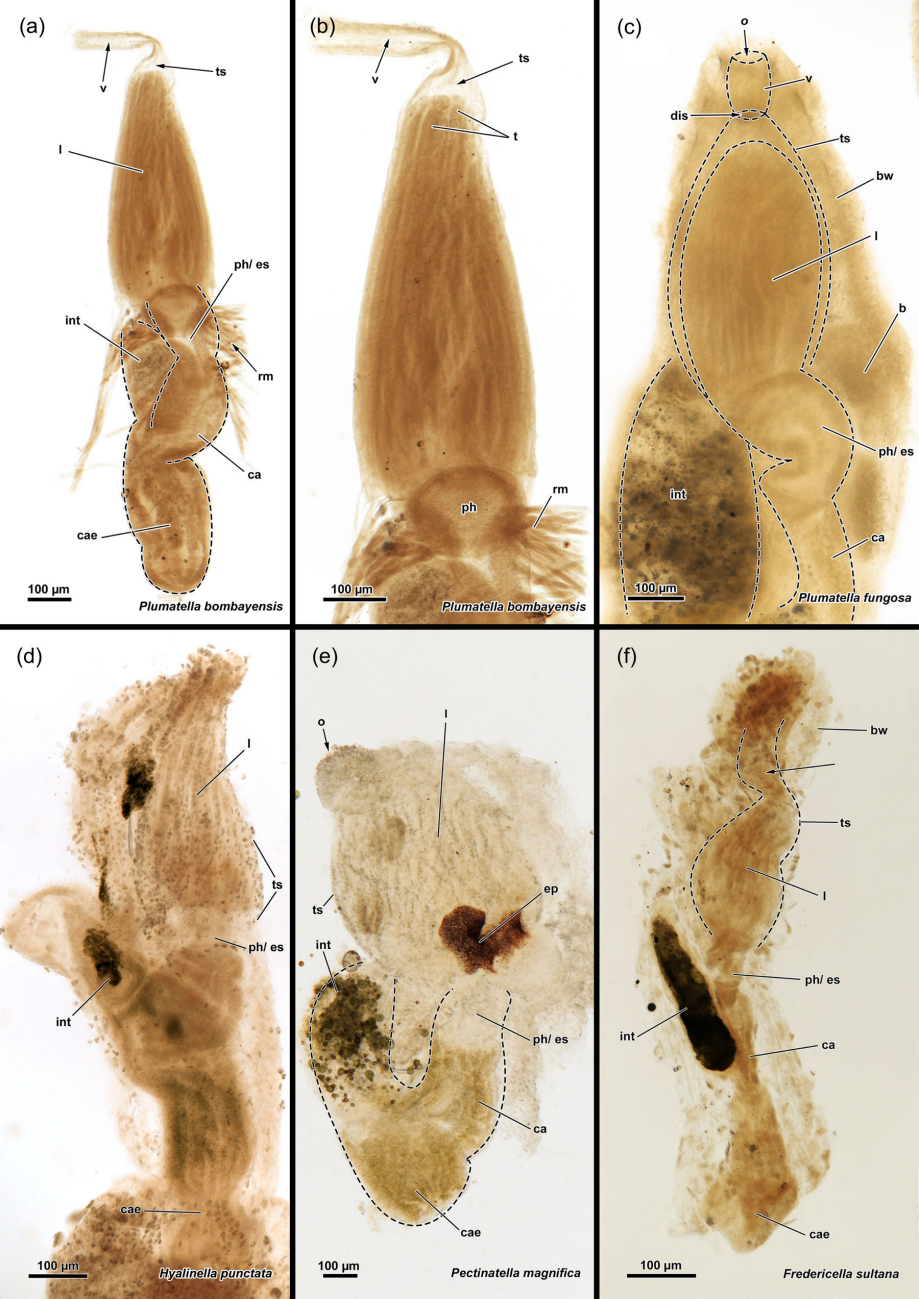
Whole mounts of various phylactolaemates. The digestive tract of the same size as the lophophore and the lophophore occupies most of the tentacle sheath (ts) in *Plumatella bombayensis* (a, b), *Plumatella fungosa* (c), *Hyalinella punctata* (d) and *Pectinatella magnifica* (e). In *Fredericella sultana* (f), the digestive tract is elongated and the lophophore is half of its size. In addition, the ts is similarly sized as the digestive tract and consequently is empty in the vestibular region (arrow). b, bud; bw, body wall; ca, cardia; cae, caecum; dis, diaphragmatic sphincter; ep, epistome; es, oesophagus; int, intestine; l, lophophore; o, orifice; ph, pharynx; rm, retractor muscle; t, tentacle; v, vestibulum.

**Figure 8:**
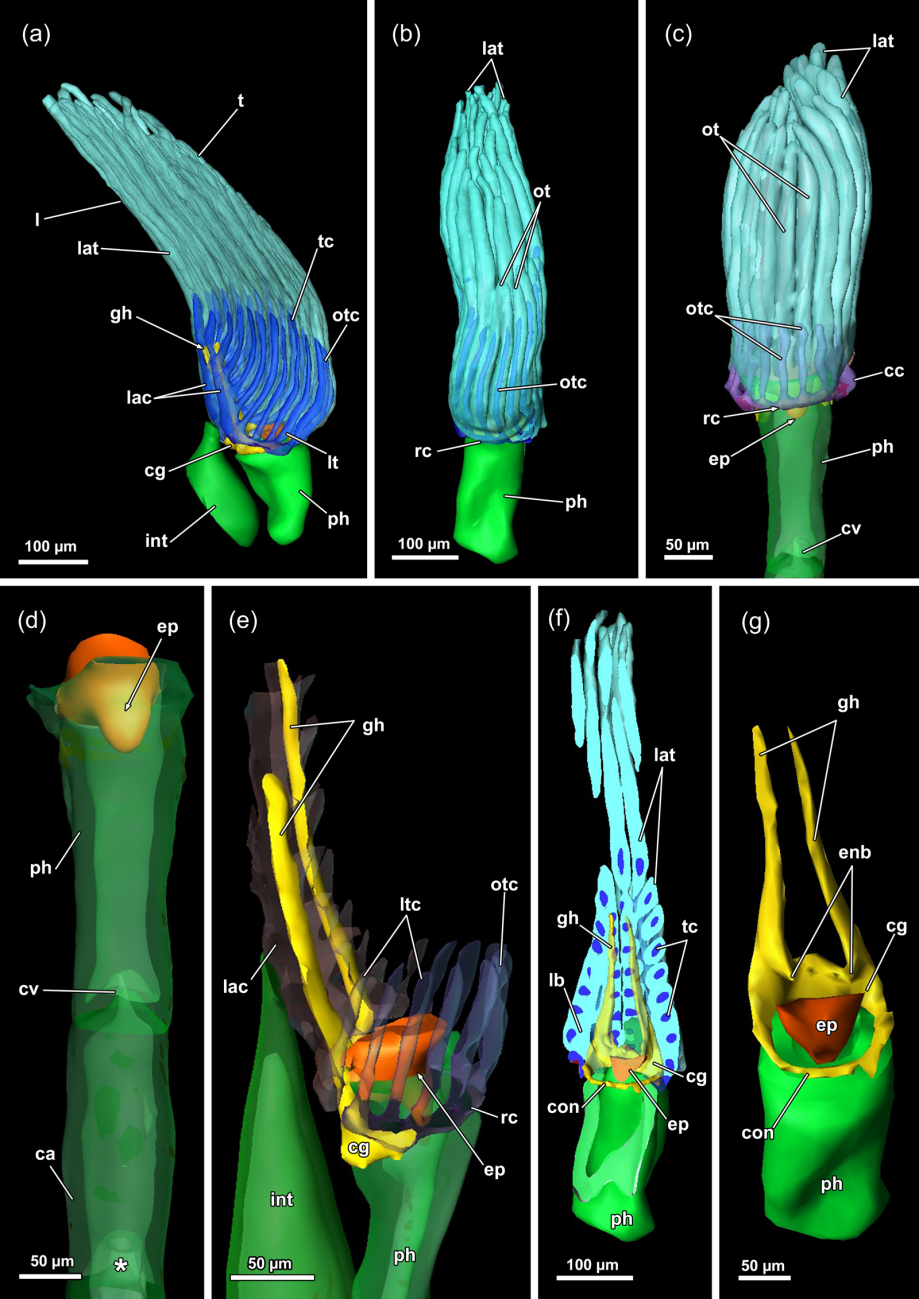
Lophophore and lophophoral base of *Hirosella fruticosa*. (a–c) Lateral (a) and frontal (b, c) views of a retracted lophophore with 40 tentacles. The tentacles of the lophophoral arms possess a coelom originating from the lophophoral arm coelom (lac), while the coelom of the lateral tentacles arises directly from the visceral coelom (a). The coelom of the oral‐most six tentacles emerges from the ring canal (rc) (b). The length of the lophophoral arm tentacles (lat) is almost twice the length of the oral tentacles (ot) (b, c). (d) A cardiac valve (cv) separates the foregut from the midgut to prevent reflux (asterisk). (e) The ring canal and lac are accompanied by corresponding projections of the nervous system: The lophophoral arms feature ganglionic horns, while the circum‐oral nerve ring extends into the ring canal. (f) Frontal section showing the circumoral‐nerve ring encircling the pharynx (ph). (g) Main areas of the central nervous system: the cerebral ganglion (cg) with the circum‐oral nerve, two ganglionic horns. Just above the epistome (ep), two small protrusions taper into the ep, the epistomial neurite bundles. ca, cardia; cae, caecum; cc, cystid coelom; con, circum‐oral nerve ring;  enb, epistome neurite bundle; gh, ganglionic horns; int, intestine; l, lophophore; lb, lophophoral base; lt, lateral tentacles; ltc, lateral tentacle coelom; otc, oral tentacle coelom; t, tentacle; tc, tentacle coelom.

**Figure 9:**
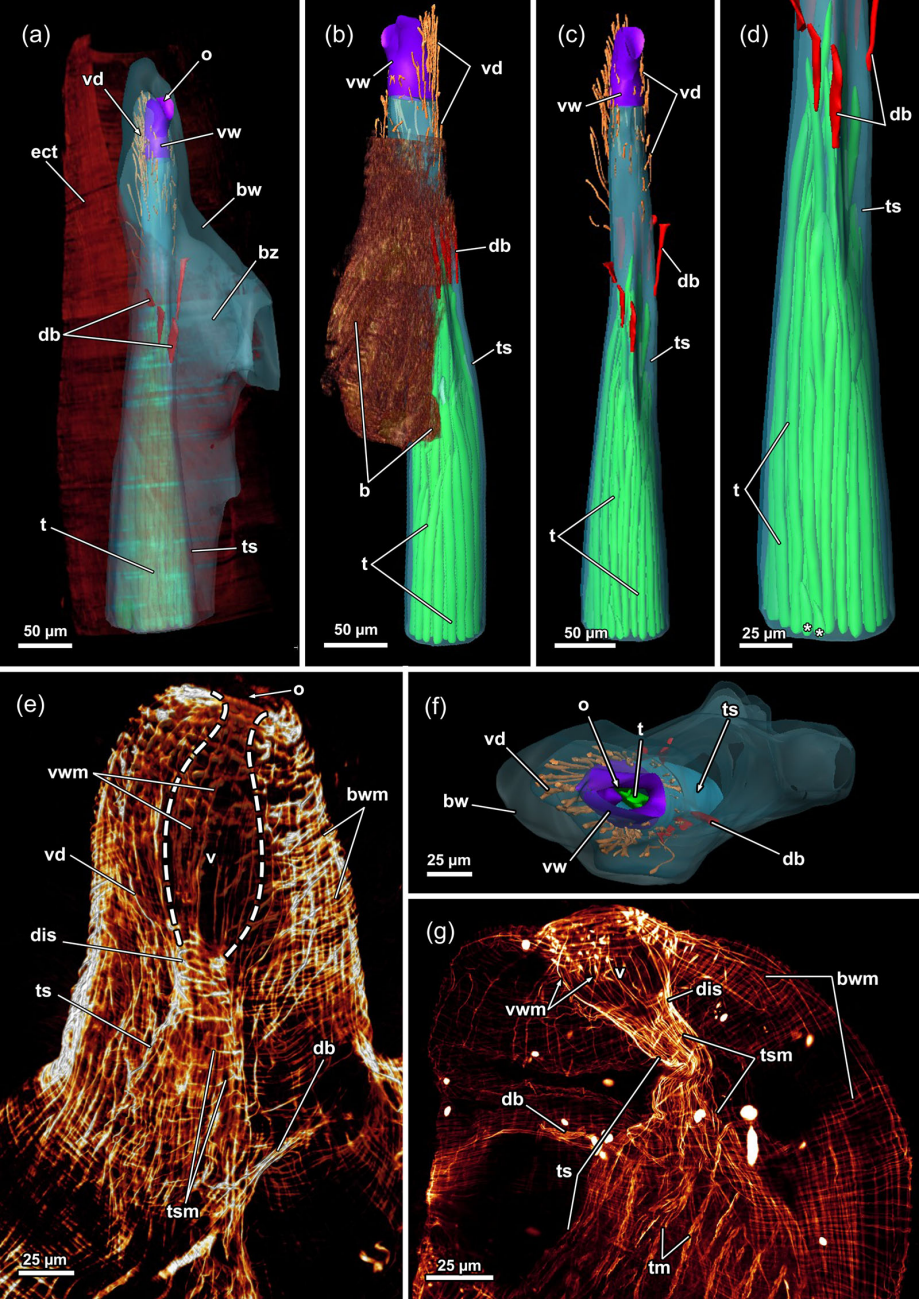
Vestibular region of *Hirosella fruticosa*. (a) The body wall (bw) invaginates at the distal end and continues as vestibular wall (vw) into the tentacle sheath (ts). (b) On the oral side, several consecutive buds form. (c) The vestibular area of the ts is not occupied by the lophophore. Vestibular dilatators insert at the vw and the vestibular area of the ts. Duplicature bands (db) insert at the vestibular end of the ts. (d) The oral tentacles terminate earlier than the remaining ones (asterisk). (e) The diaphragmatic sphincter (dis) muscle comprises several circular muscles and separates the vw from the ts. The ts include circular muscles and longitudinal muscles. (f, g) The vw has longitudinal muscles while circular muscles are absent. (f) View on the orifice showing duplicature bands and vestibular dilatators around the ts. (g) db project from the bw to the ts. b, bud; bwm, body wall musculature; bz, branching zooid; ect, ectocyst; o, orifice, t, tentacle; tsm, tentacle sheath muscles; v, vestibulum; vd, vestibulum dilatators; vwm, vestibular wall muscles.

The digestive tract starts proximal to the lophophoral base and is composed of the foregut (pharynx/oesophagus), midgut (cardia, caecum, pyloric area) and hindgut (intestine). The entire gut is elongated and about twice as long as the retracted lophophore, but approximately the same length as the tentacle sheath plus vestibular wall (Figure 4). Thus, the lophophore is rather small and fills only half to two‐thirds of the atrium (Figures 3b–h and 4b–e). The proportions of the digestive tract, the tentacle sheath and the lophophore of *H. fruticosa* are unusual, because other plumatellids (Figure 7a–d) or pectinatellids (Figure 7e) have the entire atrium occupied by the lophophore. Proportionally, the gut is of similar length as the lophophore in plumatellids and pectinatellids. Occasionally, the oral‐most tentacles are much shorter than the remaining ones in *H. fruticosa* (Figures 3e–h, 8a–c and 9c,d).

### 3.3 Lophophoral base

**Figure 10:**
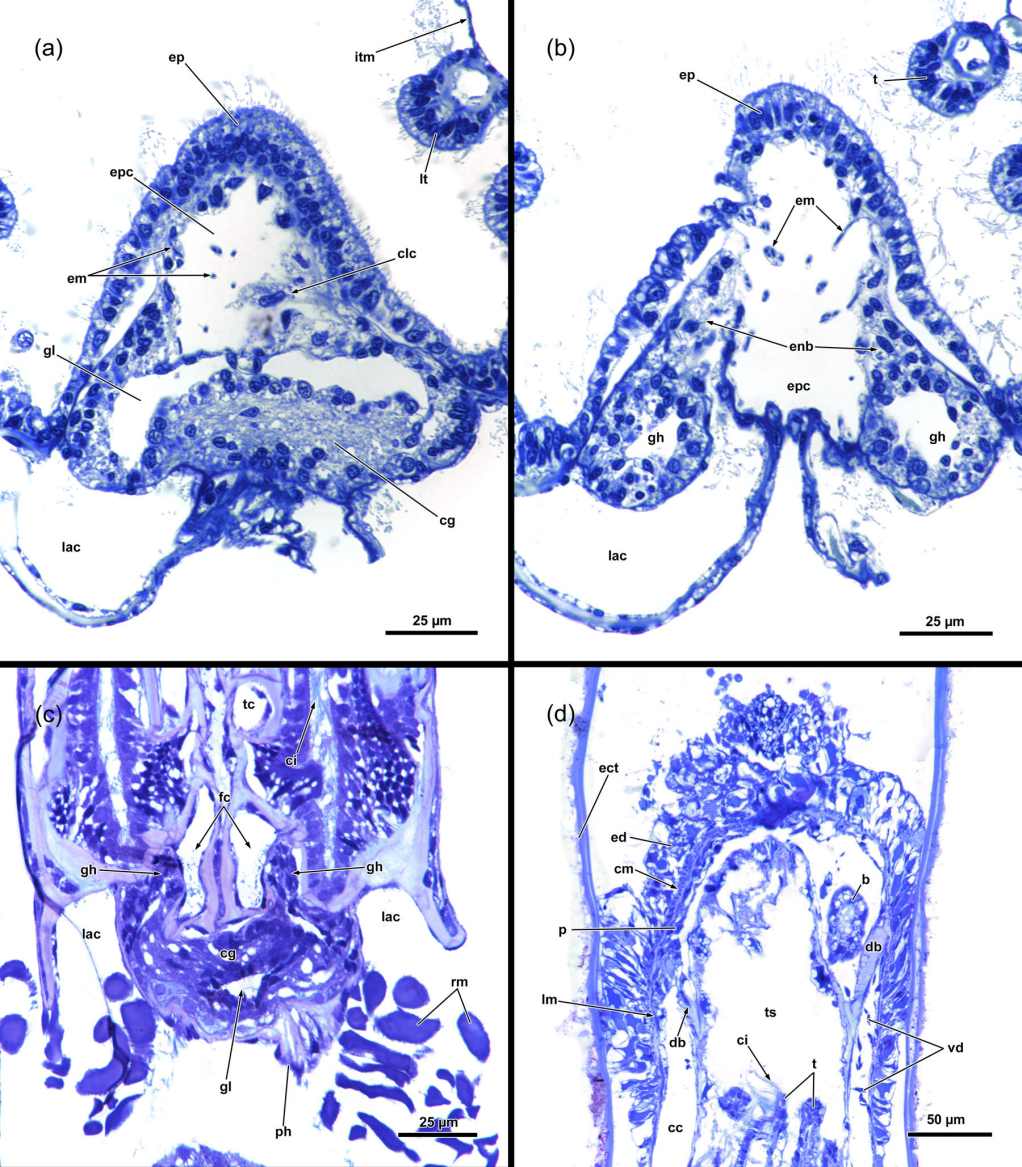
Lophophoral base and apertural area of *Hirosella fruticosa*. (a, b) Cross‐sections through the proximal (a) and distal (b) part of the epistome (ep) and central nervous system. The cerebral ganglion (cg) is located anally of the ep (a). The nervous tissue concentrates at the back of the ganglion, while the ganglion lumen is found on the oral side. Muscle fibres traverse the coelom of the ep and occasionally coelomocytes (clc) occur in the coelom (a). The cg has two ganglionic horns that project along the lophophoral arms coelom. At the lophophoral base, ep neurite bundles project from the cg into the ep (b). (c) Proximolateral retractor muscles (rm) insert close to the ganglion. The forked canal (fc) projects distal of the cg into the tentacles of the lophophoral concavity and shows some ciliation. (d) Longitudinal section through the apertural area. The body wall comprises a thick epidermal and thin peritoneal layer with circular and longitudinal muscles. Duplicature bands (db) connect the peritoneal layer of the body wall and the tentacle sheath (ts). Vestibular dilatators are present in the distal area of the zooid and overlap with the db. b, bud; cc, cystid coelom; ci, cilia; cm, circular musculature; ect, ectocyst; ed, epidermis; enb, epistome neurite bundle; epc, epistome coelom; epm, epistome muscles, gh, ganglionic horns; gl, ganglion lumen; itm, intertentacular membrane; lac, lophophoral arm coelom; lm, longitudinal musculature; lt, lateral tentacles; p, peritoneum; ph, pharynx; t, tentacle; tc, tentacle coelom; vd, vestibulum dilatators.

**Figure 11:**
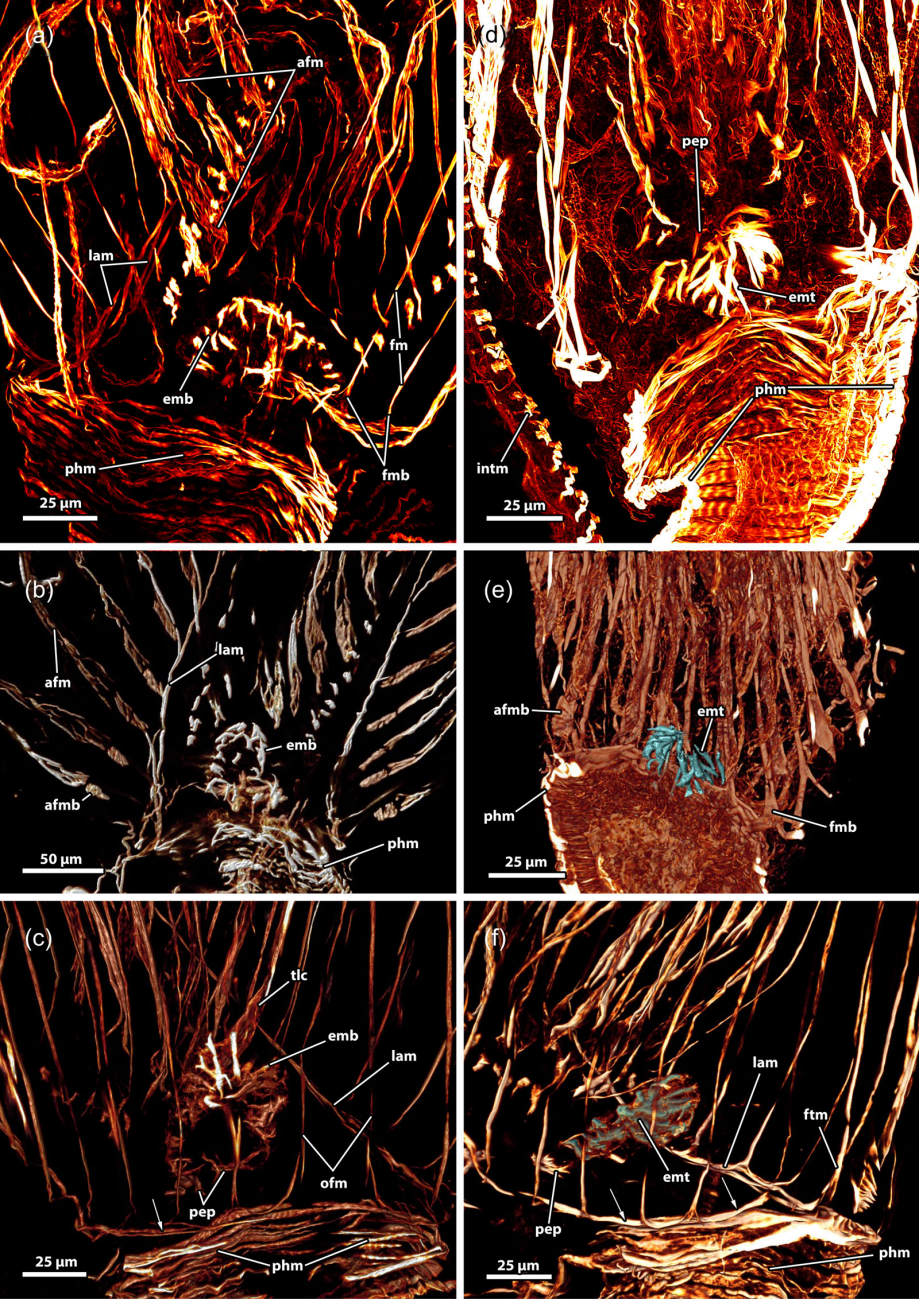
Musculature of the lophophoral base of *Hirosella fruticosa*. (a–c) The musculature of the lophophoral arms is limited to a small number of muscles. Frontal tentacle muscles are associated to the muscles of the lophophoral arms, abfrontal base muscles are not and show a gap between base and ascending tentacle muscle. The epistome has a muscular basket. (d–f) In addition to the basket‐like arrangement of muscles, the epistome also has muscle fibres traversing the coelom of the epistome, and at the proximal side of the epistome, some comparatively thin proximal epistome muscles (pep) are present. The pharynx has solely circular, striated muscles. A ring muscle is continuous from the lophophoral arm musculature and roots the frontal muscles of the oral tentacles. afm, abfrontal tentacle muscle; afmb, abfrontal tentacle muscle base; emb, epistome muscle basket; emt, transversal muscles of the epistome; fm, frontal tentacle; muscle; fmb, frontal tentacle muscle base; intm, musculature of the intestine; lam, lophophoral arm musculature; ofm, oral tentacle frontal;  phm, pharynx musculature; tlc, tentacles of the lophophoral concavity.

At the base of the lophophore, the epistome is located as a beak‐like structure above the mouth opening (Figures 5a,b,d, 8d–g and 10a,b). It features a coelomic cavity that is traversed by several muscle fibres in the oral to the anal direction (Figures 6b and 11d–f). While a muscular basket and intraepithelial epistome muscles are not identifiable on histological sections, confocal data show the presence of muscle fibres forming a basket that lines the central cavity (Figure 11a–c). The cerebral ganglion is located at the lophophoral base, proximally of the epistome (Figure 10a,b) between the pharynx and the intestine (Figures 5d, 6a and 8e–g). The ganglionic horns are large projections from the ganglion that run along the lophophoral arms (Figures 5d,e, 6b and 10a,b). The circumoral nerve ring projects orally around the pharynx (Figures 5d, 8f,g and 10). Finally, a ring canal surrounds the pharynx on the oral side of the lophophoral base and remains widely open to the remaining coelomic cavity. On the anal side, the coelomic cavity extends into the lophophoral arms.

### 3.4 Myoanatomy

#### 3.4.1 Apertural area

**Figure 12:**
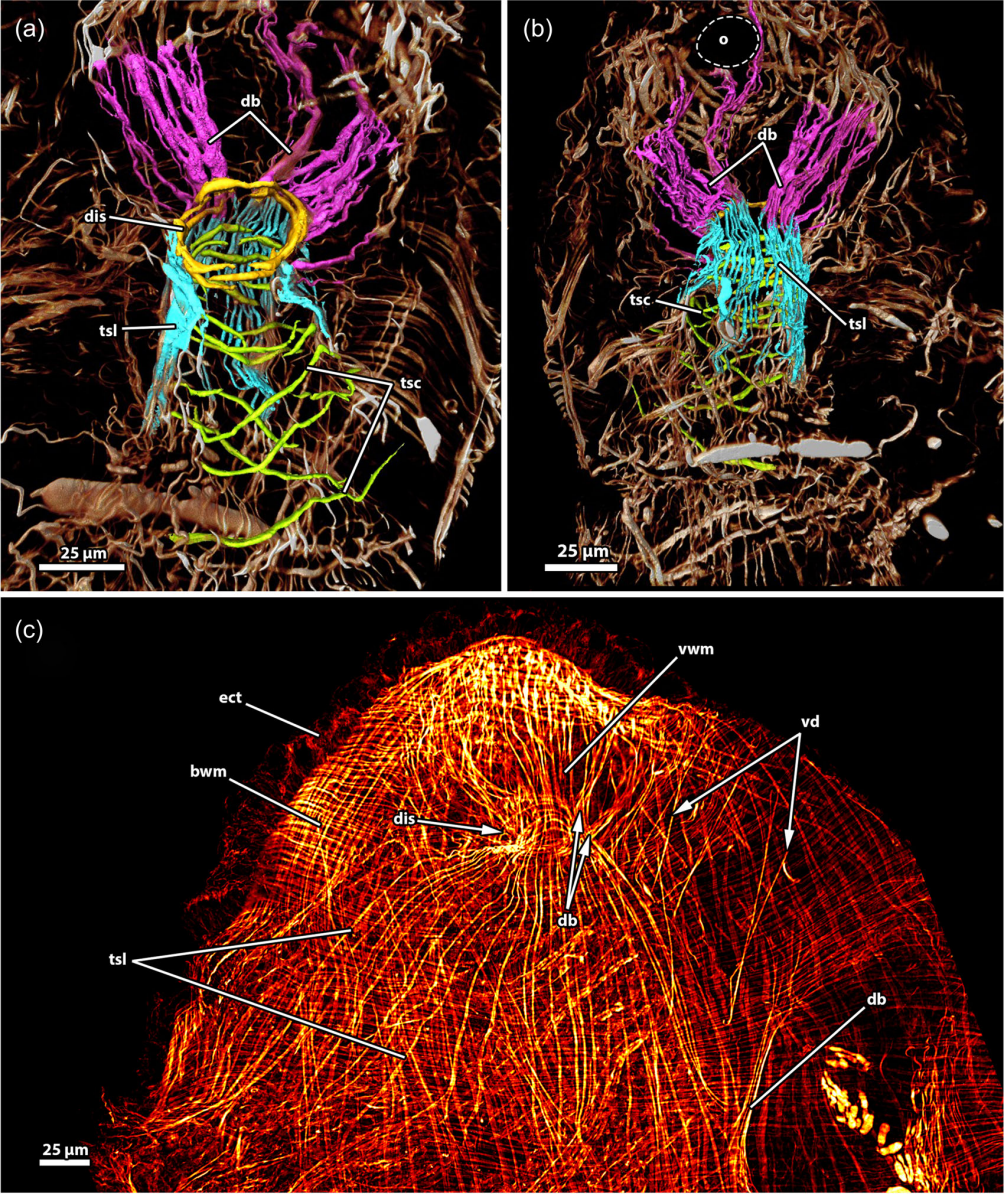
Musculature of the apertural area of *Hirosella fruticosa*. (a, b) Oral (a) and anal (b) view of the apertural area. Duplicature bands (db) project from the body wall directly to the sphincter muscle (a) and continue as longitudinal tentacle sheath muscles (tsl) in proximal direction (b). The tentacle sheath has longitudinal muscles and circular muscles that are evident in the vestibular region. (c) Orthogonal grid of body wall musculature (bwm). Underneath the latter, the longitudinal muscles of the vestibular wall are located. The vestibular wall is separated from the tentacle sheath via the diaphragmatic sphincter (dis). db insert directly at the sphincter and more proximal in the vestibular area of the tentacle sheath. The vestibular dilatators insert at the apertural region and the vestibular area of the tentacle sheath. ect, ectocyst; tsc, circular tentacle sheath muscles; vd, vestibulum dilatators; vwm, vestibular wall muscles.

**Figure 13:**
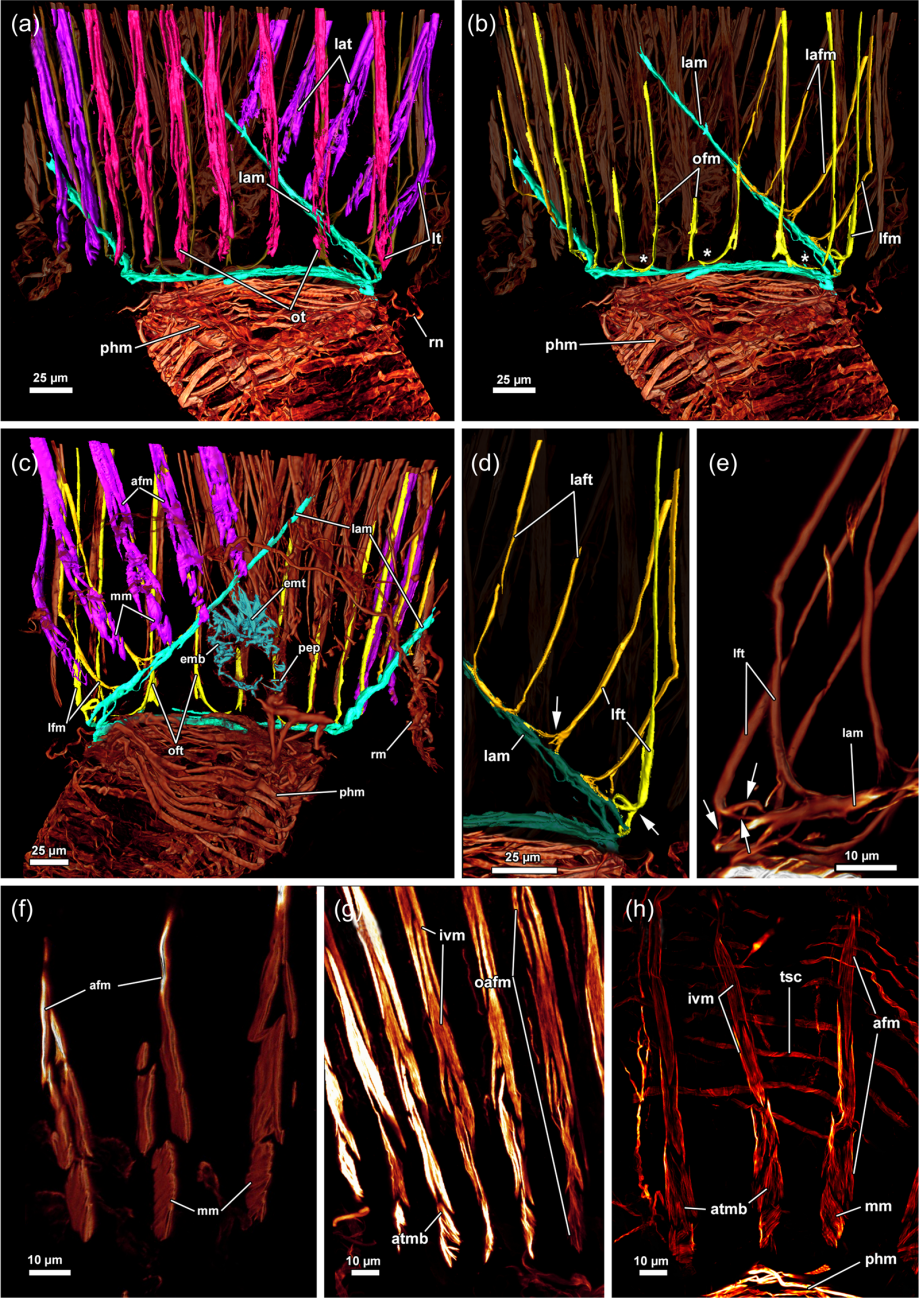
Musculature of the tentacles of *Hirosella fruticosa*. (a) View on the lophophoral base from the oral side. A ring muscle is located distal to the pharynx musculature (phm) and connects to the musculature of the lophophoral arms. The abfrontal tentacle muscles consist of comparatively small bases and ascend as rather thick abfrontal tentacle muscles in the distal direction. (b) Oral view with slim frontal tentacle muscles of the oral and lateral tentacles. Proximal, the frontal muscles of the oral tentacles are laterally interconnected by rootlets (asterisks). (c) Lateral view of the lophophoral arms. The lophophoral arm muscles comprise only a few muscle bundles. Abfrontal tentacle muscles include median muscle (mm) bands that form the base followed by comparatively thick abfrontal muscles. (d, e) Detail of the frontal muscle of the lateral and lophophoral arm tentacles. Frontal muscles are associated with the lophophoral arm muscles via three rootlets (arrows). Tentacles of the lophophoral arm show only one rootlet. (f–h) Detail of the abfrontal muscles of the lophophoral arm (f) and oral (g, h) tentacles. The base comprises a few obliquely orientated mm bands. Distal of the base, two muscle bundles fuse and ascend as tentacle muscles that appear in the form of an inverted ‘v’. The tentacle sheath includes circular muscles on the proximal side (h). afm, abfrontal tentacle muscle; afmb, abfrontal tentacle muscle base; emb, epistome muscle basket; emt, transversal muscles of the epistome; inv, inverted ‘v’ muscle; lam, lophophoral arm musculature; lfm, frontal muscles of the lateral tentacles; ofm, oral tentacle frontal muscles; pep, proximal epistome muscles; tsmc, circular tentacle sheath muscles.

The body wall musculature of *H. fruticosa* consists of an outer layer of circular muscles and an inner layer of longitudinal muscles (Figures 9f,g, 10d and 12c). The vestibular wall is separated from the tentacle sheath via a diaphragmatic sphincter muscle (Figures 9f,g and 12). The latter is hardly recognisable on histological sections and is formed by comparatively densely arranged circular muscles (Figures 9f,g and 12). Since the vestibular wall and the tentacle sheath are continuous with the body wally, circular and longitudinal muscles were expected to be found in the introverted area as well. Surprisingly, circular musculature is missing in the vestibular wall (Figures 9f,g and 12a,b). Hence, only longitudinal muscles are present in the vestibular area (Figure 9f,g), contrary to the tentacle sheath, which has longitudinal muscles and circular muscles in *H. fruticosa* (Figures 9f,g, 12a,b and 13h). The circular muscles are present starting from the diaphragmatic sphincter muscle and continuing all the way down to the proximal end of the tentacle sheath (Figures 9f,g, 12a,b and 13h). Therefore, *H. fruticosa* possesses circular musculature in the distal and proximal region of the tentacle sheath but not in the vestibular area.

Two muscle sets are associated with the apertural area: thin vestibular dilatators and duplicature bands (Figures 3b–d, 4d,e, 9a–e, 10d and 12). The former are individual muscle fibres projecting from the body wall to the vestibular area and are less abundant towards the vestibular region of the tentacle sheath (Figures 3b,c, 9a–f, 10d and 12c). The duplicature bands constitute peritoneal bands with longitudinal muscle fibres. They continue from the longitudinal body wall muscles to the longitudinal tentacle sheath muscles (Figures 3c, 9f, 10d and 12). In *H. fruticosa*, the duplicature bands connect to the tentacle sheath in the vestibular region. They occasionally are stacked, since several (up to four) duplicature bands connect directly to the area of the sphincter muscle (Figure 12). Thus, duplicature bands of *H. fruticosa* are not only arranged circularly in a plane but inserted at different levels of the apertural area.

#### 3.4.2 Lophophore and tentacle musculature

A thin muscular ring encircles the mouth opening and connects to the lophophoral arm muscles on the anal side (Figures 11c,f and 13a–d). The lophophoral arm muscles include 1–3 delicate muscle fibres that project in the anal direction from the lophophoral base and extend along the lophophoral arms (Figures 11a–c and 13a–e). Each tentacle is supplied with two ascending muscles: (1) On the side facing the inside of the lophophore, a frontal tentacle muscle ascends into each tentacle. (2) On the opposite side, facing the outside of the lophophore, abfrontal muscles (inverted ‘v’ muscles) extend into the tentacles (Figures 11 and 13). The bases of the abfrontal muscles consist of obliquely orientated muscles (median muscle bands) that are distally followed by two adjoining muscle bundles that form the inverted ‘v’ muscle. A gap is frequently encountered between the abfrontal base muscle and the inverted ‘v’ muscle (Figure 13f–h). Abfrontal muscle bases of *H. fruticosa* are not connected to the oral muscle ring or the lophophoral arm muscles. The proximal bases of the frontal muscles have up to three rootlets (Figure 13b–e). In the oral tentacles, which arise from the ring canal, these rootlets feature lateral connections (Figures 11f and 13b). The lateral connections appear associated with the circum‐oral lophophoral base ring (Figure 13b). Similar anchoring to the circum‐oral base muscle occurs in the lateral tentacles. The frontal muscle bases of the tentacles of the lophophoral arms include one or two rootlets that connect to the lophophoral arm muscles (Figure 13b–e).

#### 3.4.3 Digestive tract, retractor muscle and funiculus

**Figure 14:**
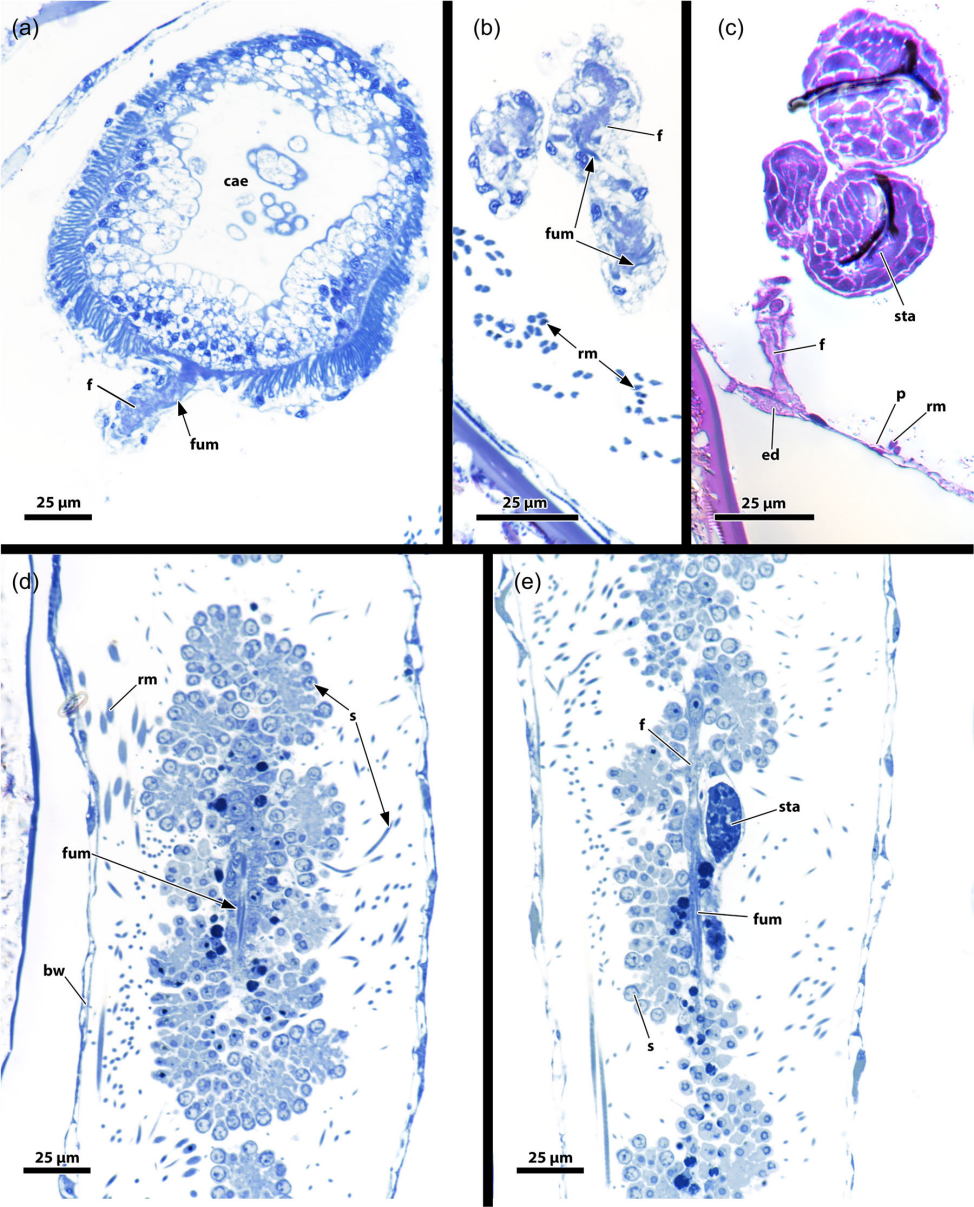
Proximal region of *Hirosella fruticosa* and *Internectella bulgarica*. (a–c) Sections of *H. fruticosa* show the proximal end of the caecum (cae). The funiculus inserts at the cae and projects to the body wall (bw). (d, e) Longitudinal sections of *I. bulgarica* show the funiculus as a thin peritoneal stand. Testes are attached to the funiculus and an anlage of a statoblast is present. The funiculus has longitudinal muscles in *H. fruticosa* and *I. bulgarica*. ed, epidermis; f, funiculus; fum, funiculus muscles; p, peritoneum; rm, retractor muscle; s, sperms; sta, statoblast anlage.

The retractor muscle of *H. fruticosa* consists of symmetric packages of thick muscle bundles (Figure 4f) that insert laterally at the lophophoral base (Figure 10c), the anal side of the tentacle sheath, several areas of the digestive tract and at the corresponding areas of the body wall (Figures 3a and 13a,c). The digestive tract of *H. fruticosa* consists of exclusively circular muscles that are most prominent in the pharynx (Figures 11 and 13a–c). At the proximal end of the caecum, the funiculus extends as peritoneal cord to the body wall. The funiculus includes longitudinal muscles (Figure 14a–c).

### 3.5 Fredericellidae

The available data on fredericellids primarily pertains to the myoanatomy of *F. sultana*. In addition, certain aspects of the general morphology of *F. sultana* and *Internectella bulgarica* have been studied to supplement the data on *Hirosella fruticosa*.

#### 3.5.1 Zooid morphology

**Figure 15:**
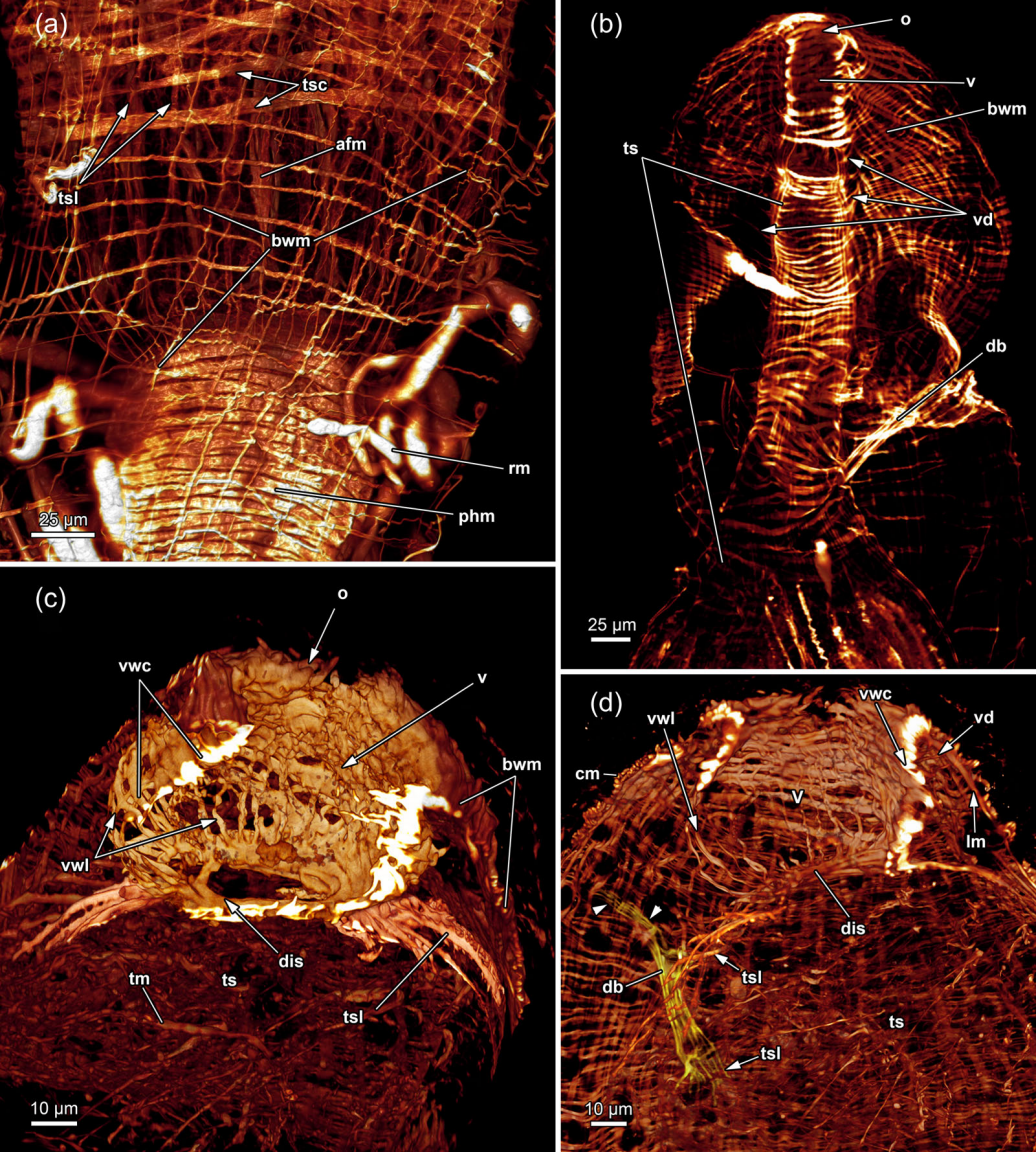
Body wall musculature (bwm) and musculature of the apertural area of *Fredericella sultana*. (a) The bwm consists of longitudinal and circular muscles. The tentacle sheath (ts) includes longitudinal muscles and prominent circular muscles. (b) The vestibular wall shows circular muscles and the ts possesses thick circular muscles and relatively slender longitudinal muscles. Duplicature bands (db) insert at the vestibular area of the ts. (c) Vestibular wall with circular and longitudinal muscles. Proximal of the vestibulum a diaphragmatic sphincter (dis) is present as a single circular muscle. (d) The circular muscles of the vestibular wall are densest close to the orifice. db connects the body wall muscles with the ts. Noteworthy, they occasionally appear continuous from the circular muscles (arrowheads) of the body wall to the longitudinal muscles of the ts. cm, circular musculature of the body wall; lm, longitudinal musculature of the body wall; o, orifice; phm, pharynx musculature; rm, retractor muscle; tsc, circular tentacle sheath muscles; tsl, longitudinal tentacle sheath muscles; v, vestibulum; vd, vestibulum dilatators; vwc, circular vestibular wall muscles; vwl, longitudinal vestibular wall muscle.

The polypide of *F. sultana* shows similar proportions as *H. fruticosa*. The digestive tract is elongated in the proximodistal axis and also the lophophore occupies only half of the tentacle sheath (Figures 7f and 15b). While the digestive tract and the tentacle sheath are also rather elongated in *I. bulgarica* (Figure 15a,b), the lophophore is large and fills the entire tentacle sheath, and therefore differs from *Fredericella* and *Hirosella* (Figures 4a–e and 15a–e).

#### 3.5.2 Apertural and tentacle sheath muscles

**Figure 16:**
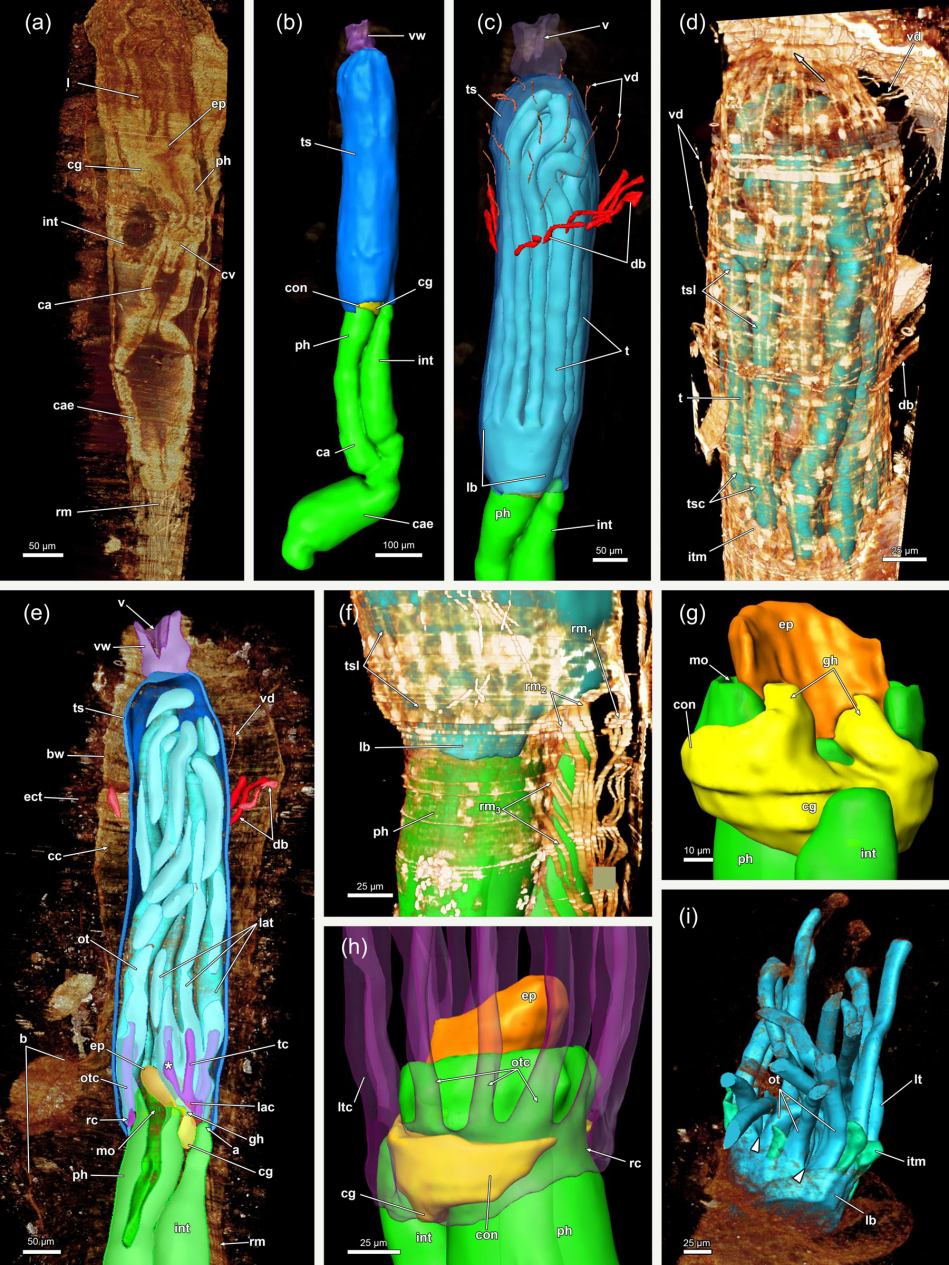
Three‐dimensional (3D) reconstruction of *Internectella bulgarica*. (a) Longitudinal section of a polypide shows the epistome (ep) as flap‐like protrusion above the anal side of the mouth. The digestive tract includes the pharynx (ph) and the cardia (ca), which are separated by the cardiac valve (cv). The caecum is spacious and long and ascends into the intestine (int). (b) 3D reconstruction of the polypide. The lophophore is almost the same length as the digestive tract. (c) The tentacles of the lophophore are notably long, extending throughout the entire tentacle sheath (ts). In the vestibular region of the ts, vestibular dilatators project from the body wall (bw) (not reconstructed) towards the former. Proximal of the vestibular dilatators several duplicature bands (db) are circularly arranged around the ts. (d) Volume renderings of the ts show longitudinal and circular muscles in the tentacle sheath and in the vestibular wall (vw) (arrows). The intertentacular membrane is visible between the tentacles. (e) db and vestibular dilatators connect the bw and ts in the vestibular region. The forked canal is located in the lophophoral concavity (asterisk). (f–h) Retractor muscle (rm) insert at various locations of the lophophore and ts. Small ganglionic horns ascend from the ganglion and terminate in proximity to the ep. On the oral side, the circum‐oral nerve ring broadly encircles the ph and results in noticeable flanks. (i) The intertentacular membrane is spanned between the tentacles proximally. Lateral of the oral‐most two tentacles, a gap is visible in the intertentacular membrane. a, anus; b, bud; cae, caecum; cc, cystid coelom; cg, cerebral ganglion; con, circum‐oral nerve ring; ect, ectocyst; gh, ganglionic horns; itm, intertentacular membrane; l, lophophore; lac, lophophoral arm coelom; lb, lophophoral base; lt, lateral tentacles; ltc, lateral tentacle coelom; mo, mouth; ot, oral tentacles; otc, oral tentacle coelom; rc, ring canal; t, tentacle; tc, tentacle coelom; tsc, circular tentacle sheath muscles; tsl, longitudinal tentacle sheath muscles; v, vestibulum; vd, vestibulum dilatators.

*Fredericella* possesses an orthogonal grid of body wall muscles (Figure 15a,b). The vestibulum features longitudinal and circular muscles in its lining in *F. sultana* (Figure 15b–d). Longitudinal and circular muscles are visible in the vestibular wall of *I. bulgarica* (Figure 16d). The diaphragmatic sphincter muscle is clearly distinguishable in *F. sultana* (Figure 15c,d). In both genera, *Fredericella* and *Internectella*, the tentacle sheath comprise longitudinal and circular muscles over its entire length (Figures 15a,b and 16d,f). At least in *F. sultana*, the circular muscles are more prominent, and the longitudinal ones are comparatively slender (Figure 15a,b).

**Figure 17:**
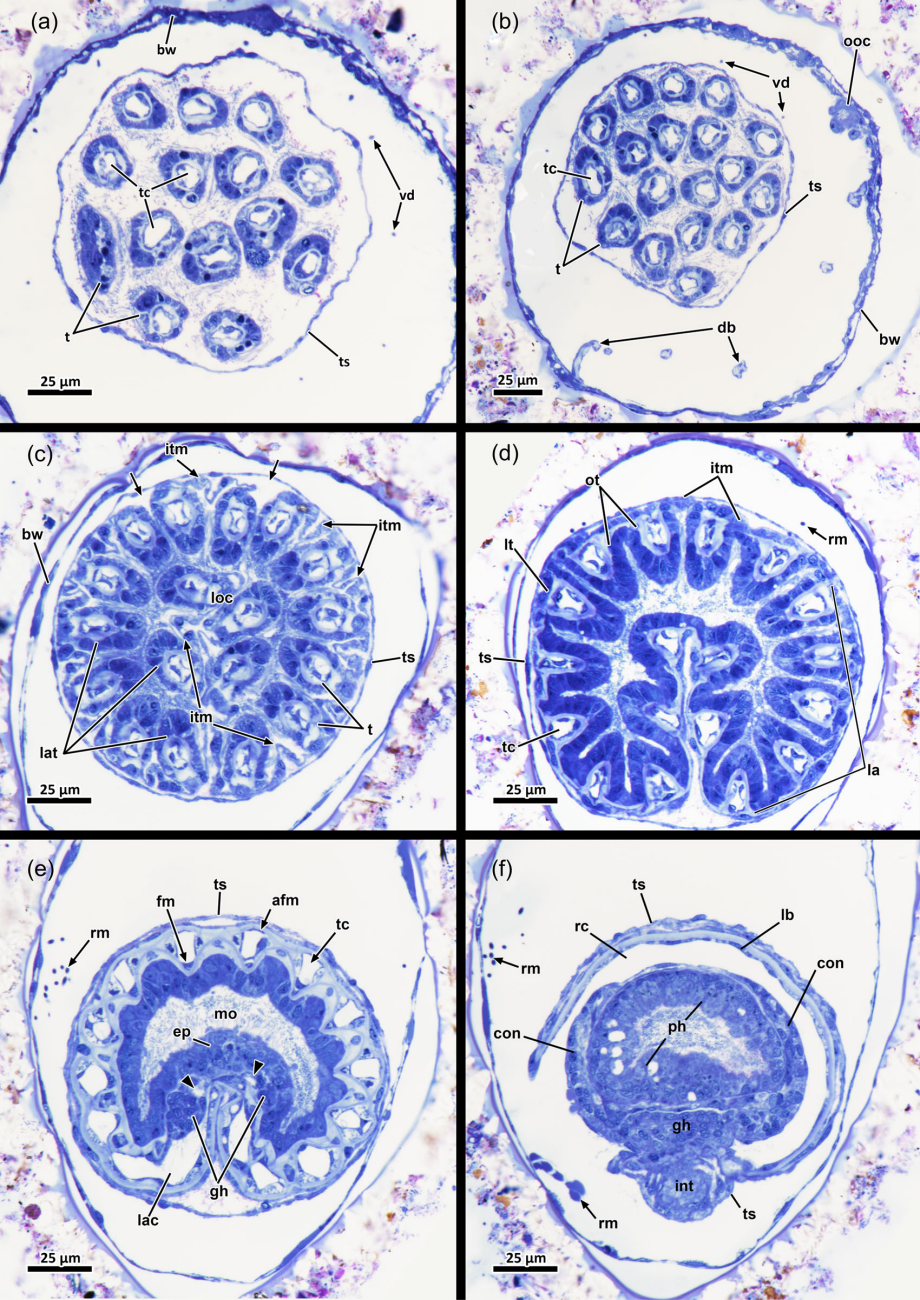
Apertural region and lophophoral base (lb) of *Internectella bulgarica*. (a) The vestibular region of the tentacle sheath (ts) is occupied by tentacles (t). Around the ts, vestibulum dilatators (vd) connect body wall (bw) and ts. (b) Duplicature bands (db) and vd are present at the same level as the ts on the oral side. (c) The intertentacular membrane (itm) is fully integrated into the epidermal layer of the t on the abfrontal side. Next to the two oral‐most t, the itm is missing. (d) When retracted, the lophophore is crescent‐shaped with well‐approachable lophophoral arms (la). (e) The epistome (ep) coelom and forked canal (arrowheads) are collapsed. Two small ganglionic horns are located anal of the supposed forked canal. Lateral, extensions of the cerebral ganglion (cg) that project as circum‐oral nerve rings in the oral direction are present. (f) A circum‐oral nerve (con) ring encircles the pharynx (ph) approximately at the level of the ring canal (rc). The latter opens to the remaining body cavity lateral of the cerebral ganglion. afm, abfrontal tentacle muscle; fm, frontal tentacle muscle; gh, ganglionic horns; int, intestine; lac lophophoral arm coelom; loc, lophophoral concavity; lt, lateral tentacles; mo, mouth; ot, oral tentacle; rm, retractor muscle; t, tentacle; tc, tentacle coelom.

All investigated fredericellids have vestibular dilatators and duplicature bands (Figures 15b,d, 16c–e and 17a). Especially the dilatators are rather inconspicuous and connect the vestibular wall and vestibular area of the tentacle sheath to the body wall (Figure 17a,b). The duplicature bands in all investigated fredericellids project from the body wall to the tentacle sheath, where they continue as longitudinal tentacle sheath muscles (Figures 15b,d, 16c–e and 17b). In contrast to *H. fruticosa*, neither *F. sultana* nor *I. bulgarica* feature duplicature bands projecting towards the diaphragmatic sphincter. Instead, they are arranged in a circular plane (Figure 16c,e). Individual scans of *Fredericella* indicate that the duplicature bands project from the circular body wall muscles and not the longitudinal ones towards the tentacle sheath (Figure 15d).

#### 3.5.3 Lophophoral base

**Figure 18:**
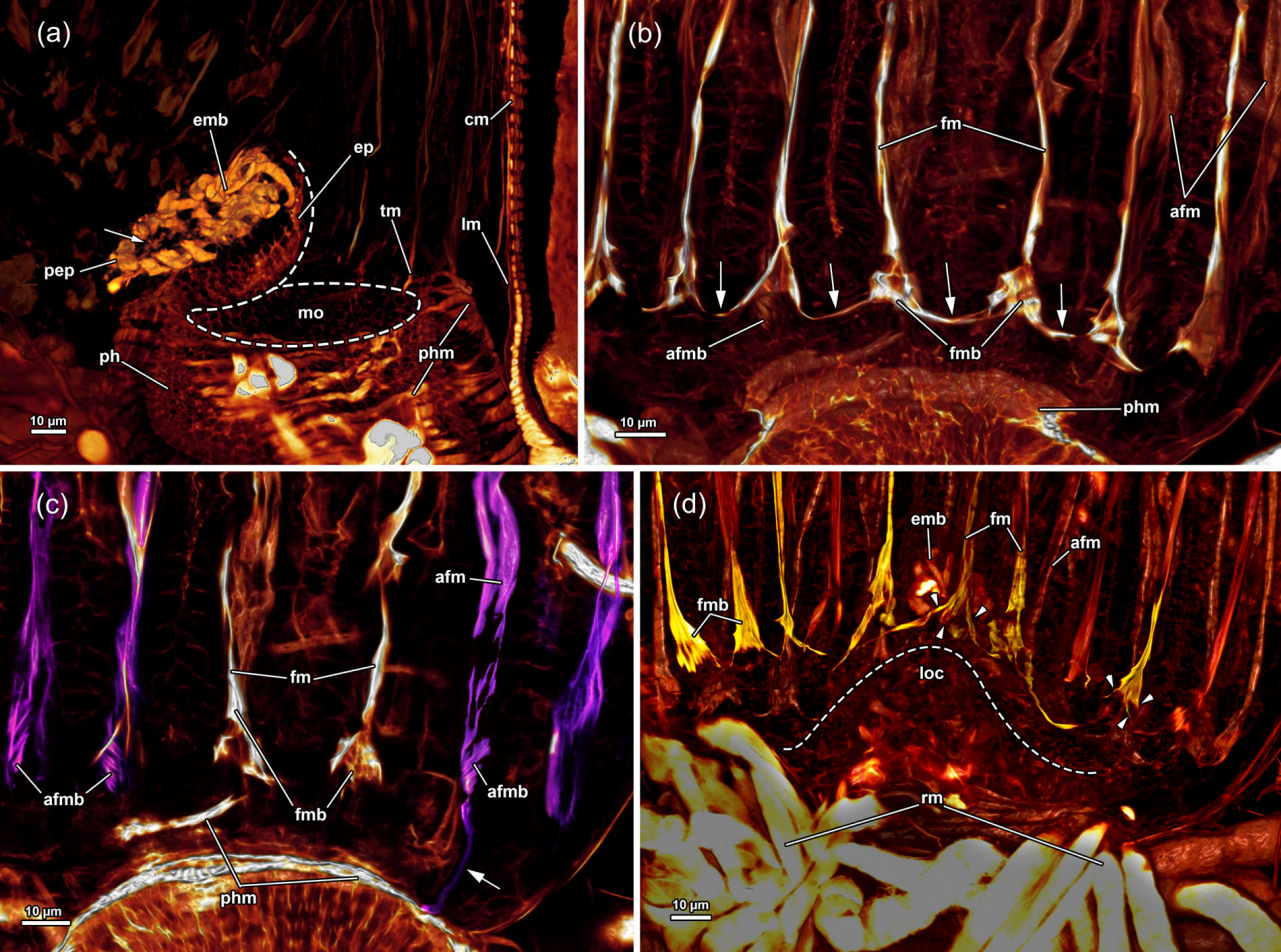
Epistome (ep) and tentacle musculature of *Fredericella sultana*, stained for F‐actin, visualised with volume renderings. (a) An oblique lateral view of the ep reveals it as a flap or knob‐like protrusion above the mouth (mo). Thick muscle bundles form a muscular basket in the epithelial lining of the ep. Additionally, proximal ep muscles are found on the anal side, while delicate muscle fibres are present at the centre of the lophophore (arrow). (b) The frontal view of the oral tentacles shows the frontal muscles ascending into the oral tentacles. Proximal, at the base of the tentacles, up to four muscle bundles intersect and form a triangular muscle base that extends as frontal tentacle muscle (fm) into the tentacles. The rootlets comprising the fontal muscle bases form continuous lateral connections between all tentacles. The frontal muscles are not connected to the pharynx (ph) musculature, nor the associated muscle ring. (c) View from the frontal side on the oral tentacle muscles (tm). The frontal muscles of the oral‐most tentacles are visible in the centre. Adjacent, abfrontal muscles of the oral tentacles ascend into the latter. The abfrontal muscle is represented by thick muscle bundles. The abfrontal muscle bases comprise some obliquely orientated muscle bands and are in general comparatively small. Proximal, the abfrontal muscle bases were occasionally found to project to the ph musculature (arrow). (d) Anal view of the tentacles of the lophophoral concavity (loc) shows the frontal muscles of the former. The central tentacle, behind the ep, shows three prominent rootlets that join and ascend as frontal muscle into the tentacle. The bases of the neighbouring tentacles include prominent rootlets as well. Tentacles of the lophophoral arms respectively in the loc form continuous lateral connections. Referred tentacles are found to have even more prominent frontal muscle bases made from several parallel muscle bands. afm, abfrontal tentacle muscle; afmb, abfrontal tentacle muscle base; cm, circular musculature of the body wall; emb, epistome muscle basket; fmb, frontal tentacle muscle base; lm, longitudinal musculature of the body wall; pep, proximal epistome muscles; phm, pharynx musculature; rm, retractor muscle.

In general, the lophophoral base in fredericellids does not differ much from *H. fruticosa*. The epistome appears as a flap or beak‐like protrusion above the mouth, which along with the general small zooid size is rather minute (Figures 16a,e,h and 18a). The small, epistomial coelom tends to collapse during the processing of the samples, which renders it impossible to address transversal muscle bundles in *I. bulgarica*. In contrast, *F. sultana* shows a muscular basket in the epithelial lining of the epistome (Figure 18a) and no indication of transversal muscle bundles. Since the data in *I. bulgarica* show a rather similar situation to *H. fruticosa*, it is conceivable that it also has a muscular basket and transversal epistome muscle.

The lophophoral base of fredericellids also includes the cerebral ganglion between the pharynx and the intestine (Figure 16e,g). While the ganglion itself is inconspicuous, its ganglionic horns are short in *I. bulgarica* and terminate after a short distance close to the epistome (Figures 16g and 17e). In contrast to these short distal projections, the lateral flanks of the circumoral nerve ring are remarkably broad (Figures 16g,h and 17f).

#### 3.5.4 Tentacle musculature and intertentacular membrane

A range of 14–17 tentacles is carried by the lophophore in the investigated fredericellids. The lophophore itself is crescent‐shaped in retracted zooids and with small lophophoral arms (Figure 17c–e). Thus, no proper lophophoral arm muscles were encountered in fredericellids (Figures 17e and 18d). Similar to *H. fruticosa*, the frontal tentacle muscles of *F. sultana* feature two to four rootlets at their bases (Figure 18b,c). In *F. sultana*, the rootlets form a triangular base with crossing fibres before ascending as frontal tentacle muscles (Figure 18b,c). In addition, they interconnect neighbouring tentacles (Figure 18b). Also, the tentacles of the small lophophoral arms show large, triangular frontal muscle bases, with the most prominent rootlets in the lophophoral concavity (Figure 18d, arrowheads). In contrast, abfrontal lophophoral muscles were comparatively small, with elongated and slim bases containing some oblique muscle bands at the proximal end (Figure 18c). Ring canal musculature was not observed in *F. sultana*. The distal pharyngeal muscles are associated with frontal lophophoral base rootlets. Occasionally, the abfrontal tentacle base muscles also show connections to the pharyngeal musculature (Figure 18c, arrow).

**Figure 19:**
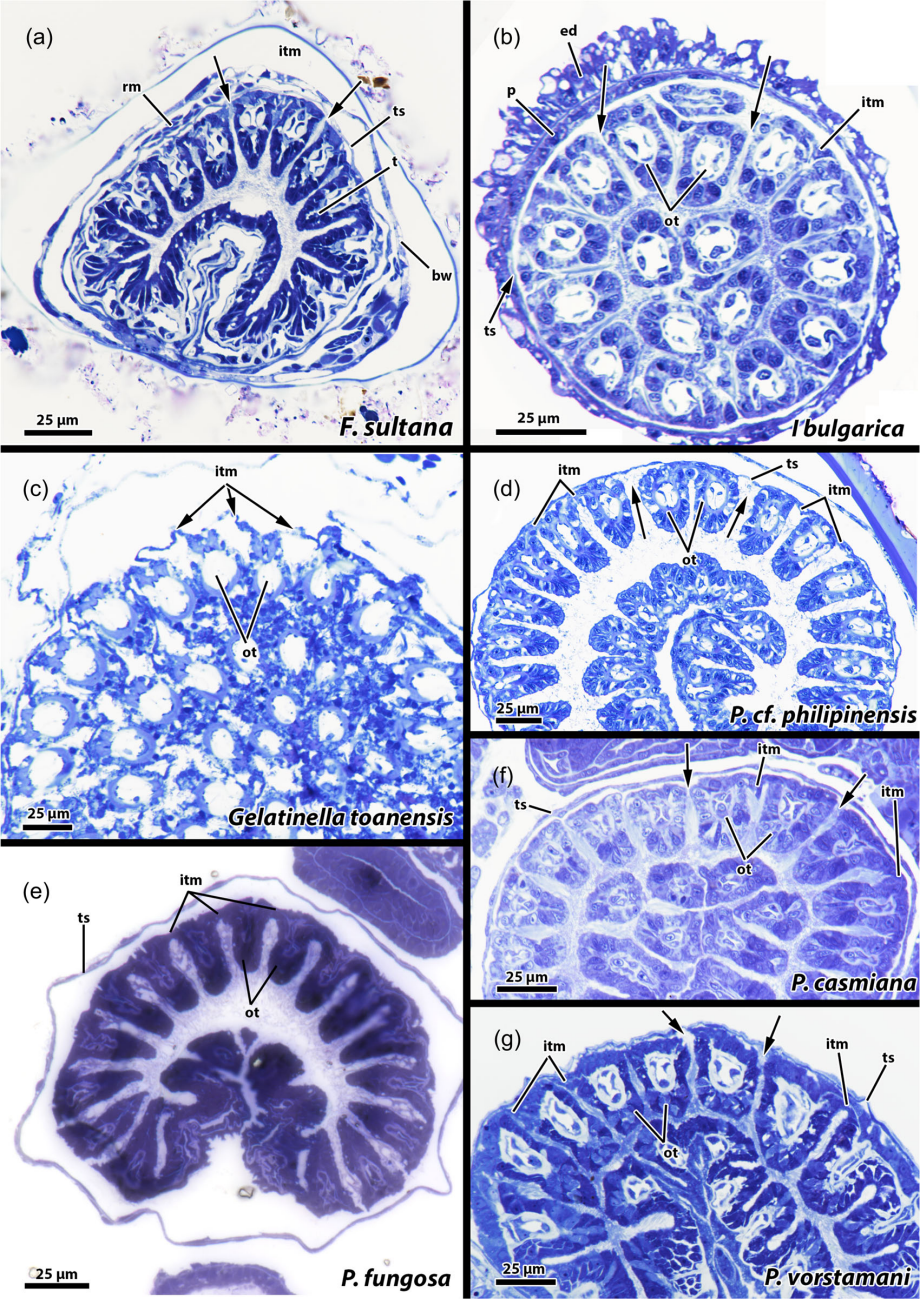
Proximal region of the lophophore of fredericellids and plumatellids. A gap is present in the intertentacular membrane lateral of the oral‐most tentacles in the fredericellids *Fredericella sultana* (a) and *Internectella bulgarica* (b). No gap is indicated in the plumatellid *Gelatinella toanensis* (c) and is present in *Plumatella* cf. *philippinensis* (d), *Plumatella fungosa* (e) and *Plumatella casmiana* (f). *Rumarcanella vorstmani* (g) shows a gap next to one tentacle, but not adjacent to the most oral tentacles (ot) and is hence inconclusive in this aspect. bw, body wall; ed, epidermis; itm, intertentacular membrane; p, peritoneum; ts, tentacle sheath.

The intertentacular membrane is connecting the proximal area of adjacent tentacles (Figures 16d,i, 17c,d and 19a,b). A gap in the membrane is present in the oral‐most tentacles in both investigated fredericellids (Figures 16i, 17c and 19a,b). In contrast to *H. fruticosa*, no medio‐abfrontal lamella or peg attaches the membrane to the tentacles; instead, the attachment is medio‐continuous with the lateroabfrontal epithelial lining of each tentacle (Figure 17c,d).

A comparison to plumatellids shows a similar situation as in the fredericellids (Figure 19c–g). Moreover, a gap next to the oral‐most tentacles is also present in c.f. *P. philipinensis*, *P. casmiana* and possibly *Rumarcanella vorstmani* (Figure 19d,f,g), whereas the plumatellids *P. fungosa* and *Gelatinella toanensis* probably lack this gap (Figure 19c,e).

#### 3.5.5 Digestive tract, retractor muscle and funiculus

Apart from its elongated form, the digestive tract is inconspicuous and has only circular musculature (Figures 15a, 16a,b and 18a–c). The retractor muscles insert at different parts of the digestive tract, at the anal side of the lophophore and tentacle sheath (Figure 16f). In addition, retractor muscles insert at the oral side of the retracted lophophore, more precisely, proximo‐orally at the tentacle sheath in *I. bulgarica* (Figure 17d–f). The funiculus lies proximal to the digestive tract. In *I. bulgarica* lots of sperms as well as a statoblast anlage were encountered on the funiculus. The funiculus of *I. bulgarica* includes longitudinal muscle fibres (Figure 14d, e).

### 3.6 Systematic account

Class: Phylactolaemata Allman, 1856; family Hirosellidae fam. nov.; genus *Hirosella* gen. nov.; type species *Hirosella fruticosa* comb. nov.; etymology: in honour of our bryozoologist colleague Masato Hirose, who greatly contributed to freshwater bryozoan research.

#### 3.6.1 Diagnosis

Colony erect with only a few, early astogenetic zooids adherent and creeping on the substrate. Cystids of primary branches with numerous stumps are responsible for the typical serrated appearance. Lophophore horseshoe‐shaped with 32–55 tentacles, filling only about two‐thirds of the atrium in a retracted condition. Gut highly elongated, particularly fore‐ and hindgut. Floatoblasts and sessoblasts are highly elongated, at least twice as long as broad. Sessoblasts with well‐developed annulus showing uninflated float. Duplicature bands with multiple insertion areas, tentacle sheath and diaphragmatic sphincter. Vestibular wall with longitudinal muscles only. Intertentacular membrane showing lamellated/pegged attachment to tentacles, larvae with only one functional polypide.

## 4 DISCUSSION

### 4.1 Colony morphology and phylogenetic considerations

Early phylactolaemate research already indicated that *Hirosella fruticosa* is easily mistaken for *Fredericella* and the fact that both often co‐exist in the same habitat renders identification at first sight even more difficult (Allman, 1856; Borg, 1941; Braem, 1890; Wiebach, 1954). In theory, *H. fruticosa* is easily distinguishable from any fredericellid by the presence of a horseshoe‐shaped lophophore, contrary to the circular one in fredericellids (Du Bois‐Reymond Marcus, 1946b; Gruhl & Bartolomaeus, 2008; Marcus, 1926; Shunkina et al., 2015). *Hirosella fruticosa* produces elongated floatoblasts while fredericellids have bean‐shaped piptoblasts, which lack an annulus (Gruncharova, 1971; Wood, 1983, 2010, 2015; Wood & Backus, 1992; Wood & Okamura, 2005). However, statoblasts are not always present and lophophores are difficult to distinguish or retract when specimens are examined in the field. Hence, it is not surprising that *H. fruticosa* has often been confused with *Fredericella*. This is largely based on similar colony morphology. Fredericellids form erect, branching colonies, which is relatively unusual among phylactolaemates, except for *H. fruticosa* and *Plumatella osburni* (Toriumi, 1952). Under certain conditions some plumatellids form erect growing branches (e.g., Allman, 1856; Wood, 2015; Wood et al., 2006), but different from *Fredericella* and *H. fruticosa*.

Despite their often almost identical appearances, some minor differences are present between fredericellids and *Hirosella fruticosa*: the main branches of *Fredericella* tend to creep on the substrate and form erect buds that detach from the latter (Braem, 1890; Gruncharova, 1971; Wood & Okamura, 2005). In contrast, colonies of *H. fruticosa* are only attached to the substrate at their origin (Allman, 1856; Toriumi, 1954; present study). The erect colonies with distinctly larger zooidal size and shrubby appearance are decisive characters of *H. fruticosa* (Allman, 1844, 1856; Kraepelin, 1887; Toriumi, 1954). In addition, *H. fruticosa* forms cylindrical cystid tubes that dilate at the distal end (Allman, 1844; Toriumi, 1954, this study).

It is not surprising that a closer relationship between *Hirosella* and *Fredericella* has repeatedly been suggested (Braem, 1908; Toriumi, 1956). The most recent phylactolaemate phylogeny confirms that *H. fruticosa* is not a plumatellid (Saadi et al., 2022), but instead clusters as sister taxon to Cristatellidae and Pectinatellidae. Furthermore, this so‐called ‘PCP’ clade (including *H. fruticosa*), represents the sister taxon to Fredericellidae. Consequently, *H. fruticosa* is indeed more closely related to *Fredericella* than *Plumatella*.

Besides the general colony morphology, the present study documented other aspects that support the phylogenetic placement of *Hirosella*. As mentioned above, *H. fruticosa* frequently features a serrated cystid on its oral side (see also Toriumi, 1954; Wiebach, 1954). This was reported as a common characteristic for each zooid in *H. fruticosa* and even from ancestrulae onwards (Toriumi, 1954). The serrated appearance is a result of the budding process, which in all phylactolaemates, except for *Stephanella*, is restricted to the oral side of zooids (Braem, 1890, 1897, 1908; Jebram, 1973; Mukai, 1990; Oka, 1908; Schwaha et al., 2020). However, as consecutive buds form, only a few of them remain attached to their maternal zooid and most of them break off, resulting in a serrated appearance (Toriumi, 1954; Wiebach, 1954; this study). The fragmented colony pieces are considered to disperse and eventually found new colonies (Toriumi, 1954; Wood, 2015). Besides *H. fruticosa, F. sultana* can also have a slightly serrated appearance owing to so‐called proliferation buds. However, these buds do not occur frequently in *F. sultana* (Toriumi, 1954). *Hirosella fruticosa* zooids have approximately 15 remains of proliferation buds, whereas *F. sultana* zooids have a maximum of five such remains (Wiebach, 1954).

Another interesting character is the keel on the oral cystid. It is a variable character that can be present and absent within the same species like, for example, *P. repens* (Braem, 1890; Hirose & Mawatari, 2011a; Wood, 2015; Wood & Okamura, 2005). Nevertheless, a keel has been described for several plumatellids, for example, *P. emarginata*, *P. fungosa*, *P. repens, P. javanica* (Allman, 1856; Braem, 1890; Hancock, 1850; Hirose & Mawatari, 2011a, 2011b; Smith & Wood, 1995; Wood & Okamura, 2005) and fredericellids (Allman, 1856; Du Bois‐Reymond Marcus, 1946a; Wood & Okamura, 2005). Despite the variable nature of this character, every single report of *H. fruticosa* features an at least moderately developed keel (Allman, 1856; Braem, 1890; Kraepelin, 1887; Toriumi, 1954; Wood & Okamura, 2005; present study).

### 4.2 Zooid morphology

In addition to aspects of the general morphology, differences occur also on the zooidal level. The proportions of the tentacle sheath, lophophore and digestive tract in *H. fruticosa* differ from other plumatellids and resemble those in fredericellids (mostly *F. sultana* [Braem, 1908; Klymkiw & Wanninger, 2019; this study]). Hence, this never investigated character unites *H. fruticosa* with fredericellids but also distinguishes it from plumatellids. Particularly the caecum and intestine are elongated in *Fredericella* and large compared to the lophophore. Moreover, *F. sultana* has previously been depicted with a lophophore, that is, smaller than the tentacle sheath, which leaves plenty of space in its distal area (Braem, 1908)—unlike plumatellids (Braem, 1890; Hancock, 1850). Notably, the fredericellid *I. bulgarica* shares the elongated gut proportions (Wood et al., 2006), but in regard of the lophophore shows a typical plumatellid condition. In this respect, *I. bulgarica* fits its original genus etymology and shows a mosaic of fredericellid and plumatellid traits. Unfortunately, there are no transcriptomic data available yet, which could clarify this species' position and allow for better character evolution discussion.

Apart from colony morphology and zooid morphology, an ontogenetic character is shared by *H. fruticosa* and fredericellids: sexually produced mantle larvae in plumatellids and other families include two successively formed polypides (Bibermair et al., 2021; Braem, 1897). Fredericelid larvae include only one polypide (Braem, 1908; Gruhl, 2010), which is also the case in *H. fruticosa* (Allman, 1856; Schwaha, personal observation).

### 4.3 Lophophore

The number of tentacles is variable among different genera but also within a species or even colony (Braem, 1890; Hyatt, 1866; Kraepelin, 1887). Different families generally show a different range of tentacles, which corresponds to the size of the lophophore. Previous studies counted at least 50 tentacles in *H. fruticosa* (Braem, 1897) or provided a range of 32–55 tentacles (Toriumi, 1954). At least 40 tentacles were counted in the present study of *H. fruticosa*, which fits well with the documented range (Toriumi, 1954). With a similar general size of the lophophore, *H. fruticosa* fits well in the range of tentacle numbers of other plumatellids (20–65 [rarely up to 80], Hirose & Mawatari, 2007, 2011a, 2011b; Hyatt, 1866; Lacourt, 1968; Wood & Okamura, 2005). With the miniaturisation of the fredericellid zooid and its lophophore, tentacle numbers vary only from 15 to 28 (Allman, 1856; Braem, 1890; Hirose & Mawatari, 2011a; Hyatt, 1866; Kraepelin, 1887; Wood et al., 2006; Wood & Okamura, 2005). The oral tentacles are occasionally shorter than the remaining ones in *H. fruticosa*. Size differences of tentacles are known in some gymnolaemates (Winston, 1978) and are related to feeding behaviour (Shunatova & Ostrovsky, 2002). However, no recent data for phylactolaemates mentioned heteromorphic tentacles. Some early accounts postulated the oral tentacles to be the longest tentacles in *P. fungosa* (Nitsche, 1868) and *Cristatella mucedo* (Hyatt, 1866), which was recently reported to be more variable in length (Tamberg & Shunatova, 2017; Tamberg et al., 2014).

### 4.4 Intertentacular membrane

The intertentacular membrane is a phylactolaemate‐specific character (Braem, 1890; Schwaha et al., 2020). Early investigations noted a gap in the intertentacular membrane next to the oral‐most pair of tentacles in plumatellids and fredericellids, and only recently a study confirmed oral gaps or slits in the intertentacular membrane in *F. sultana* and *P. fungosa* (Tamberg & Shunatova, 2017). This gap has been noted to be absent in the monotypic cristatellids (Braem, 1890; Gawin et al., 2017; Kraepelin, 1887; Verworn, 1888) and comparative data for the other families and all other plumatellids remains fragmentary and not properly documented. At least the plumatellids *P*. cf. *philippinensis*, *P. casmiana* and possibly *Rumarcanella vorstmani* possess this gap (this study) whereas *Gelatinella toanensis* perhaps lack this character. Confirmed phylactolaemate taxa with oral intertentacular gaps are thus fredericellids, several plumatellids and *Hirosella* (Braem, 1890; Klymkiw & Wanninger, 2019, personal observation). Consequently, this character presumably evolved just once at the base of the clade comprising the bulk of all Phylactolaemata except Lophopodidae and Stephanellidae and was lost in Pectinatellidae and Cristatellidae; possibly also in some plumatellids. In addition, attachment of the intertentacular membrane is on the lateroabfrontal margin of each tentacle in fredericellids, lophopodids, pectinatellids (monotypic) and plumatellids (Bibermair et al., 2022; Braem, 1890; Hyatt, 1866; Mukai & Oda, 1980; Nitsche, 1868; Rogick, 1935), whereas it is attached to individual tentacles via a lamella/peg in cristatellids (Braem, 1890; Nitsche, 1868) and *Hirosella* (this study; Braem, 1890; Nitsche, 1868). Interestingly, the early‐branching and also monotypic Stephanellidae show both types of attachment forms of the intertentacular membrane (Schwaha & Hirose, 2020).

### 4.5 Myoanatomy

Comprehensive studies on the myoanatomy of phylactolaemate families are available from recent studies: Stephanellidae (Schwaha & Hirose, 2020), Lophopodidae (Bibermair et al., 2022), Cristatellidae (Schwaha, 2019), Pectinatellidae (Gawin et al., 2017), Fredericellidae and Plumatellidae (Schwaha & Wanninger, 2012). The general myoanatomy of *H. fruticosa* is similar to the rest of the phylactolaemates. Detail of major differences will be listed below.

#### 4.5.1 Body and vestibular wall musculature

The body wall of *Hirosella fruticosa* includes a circular and a longitudinal layer of musculature, which appears to be the phylactolaemate ground pattern since only *Pectinatella magnifica* and *Lophopus crystallinus* happen to feature a third, diagonal layer of muscle fibres in some areas of the cystid (Gawin et al., 2017; Marcus, 1934). At the orifice, the body wall enters the vestibular wall, which represents a duplicature of the former (Braem, 1890; Mukai et al., 1997). When the polypide is retracted, the vestibular wall continues proximally into the tentacle sheath and is separated from the latter via a diaphragmatic sphincter in all phylactolaemates (Bibermair et al., 2022; Gawin et al., 2017; Mukai et al., 1997; Rogick, 1937; Schwaha, 2019, 2020a; Schwaha & Hirose, 2020; Schwaha & Wanninger, 2012). In general, this sphincter has smooth subepidermal, circular muscle fibres in the proximal part of the vestibular wall (Mukai et al., 1997). While this sphincter muscle is often prominent and well approachable in some species, for example, lophopodids (Bibermair et al., 2022; Mukai et al., 1997) or plumatellids (Schwaha & Wanninger, 2012), it can also be barely distinguished from the surrounding circular muscles as, for example, in Stephanellidae (Schwaha & Hirose, 2020). Especially owing to the lack of circular muscles in the vestibular wall, the sphincter is easily differentiable in *H. fruticosa* and forms a dense arrangement of fibres at the vestibular end of the tentacle sheath. Although not as dense, a clear sphincter comprised of a short ring is also present in *F. sultana*.

**Figure 20:**
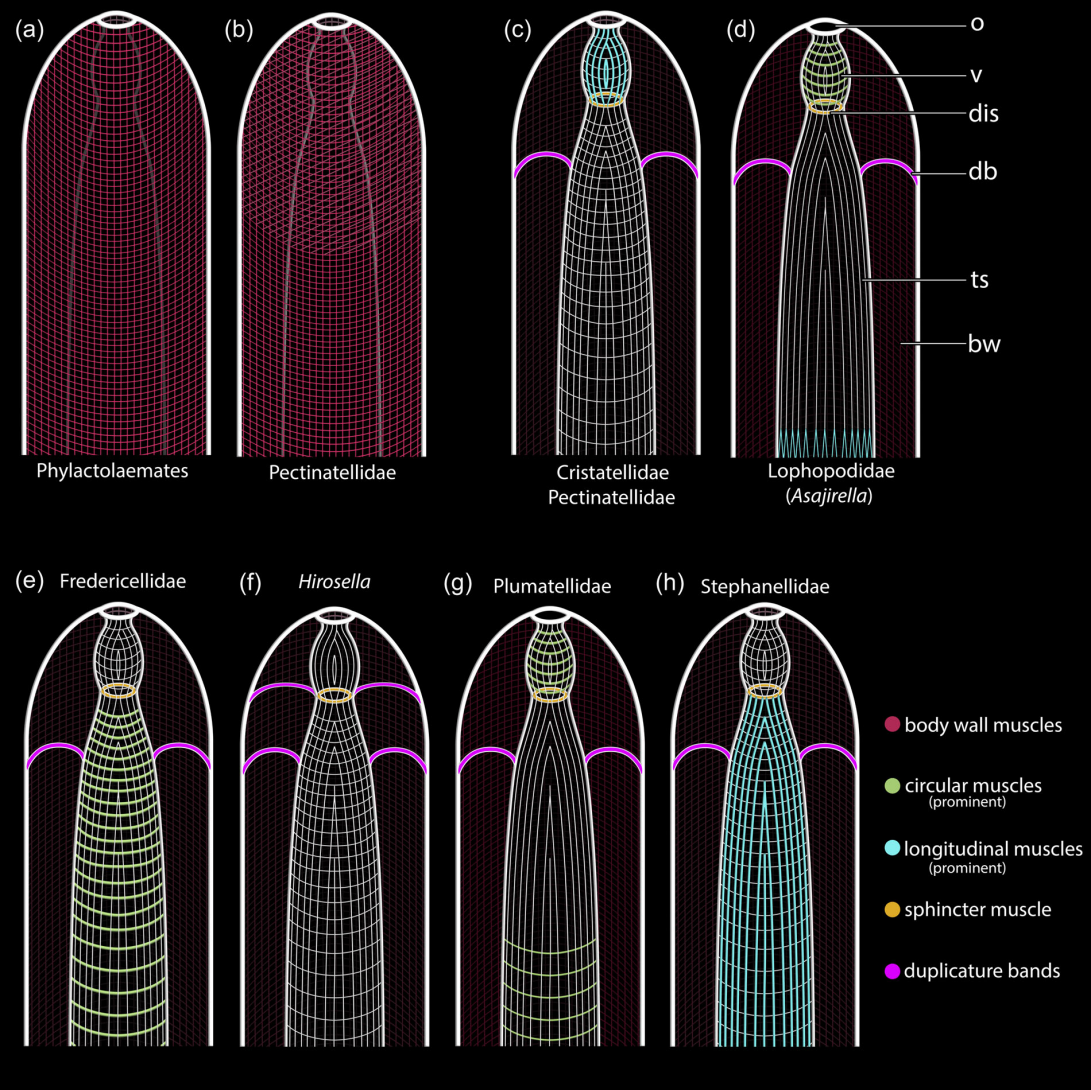
Schematic drawings of the body wall (bw) musculature and variation of apertural muscles among phylactolaemate families. (a) The ground‐pattern of the phylactolaemate bw muscles comprises an orthogonal grid of circular and longitudinal bw muscles. (b) Pectinatellidae and *Lophopus* possess a third layer of diagonal muscles. The orthogonal musculature is continuous from the bw into the vestibular wall and the tentacle sheath (ts) muscles. Each phylactolaemate clade differs from this scenario and shows specific aberrations. (c) Cristatellids and pectinatellids have circular and longitudinal muscles in the vestibular wall and ts, with the longitudinal muscles of the vestibulum (v) more prominent. (d) Lophopodids have pronounced circular muscles in the vestibular wall and lack circular musculature in the ts. The longitudinal ts musculature bifurcates proximally. (e) Fredericellids have circular and longitudinal muscles in the vestibular wall and the ts, with thicker longitudinal muscles in the ts. (f) *Hirosella fruticosa* lacks longitudinal vestibular muscles and has longitudinal and circular muscles in the vestibular and the lophophoral region of the ts. (g) Plumatellids have pronounced circular and comparatively thinner longitudinal muscles in the vestibular wall. The ts features longitudinal muscles and circular muscles. The latter is restricted to the proximal region. (h) Stephanellids have longitudinal and circular muscles in the vestibular wall and the ts, with the longitudinal ones being thicker in the ts. Duplicature bands (db) are arranged in a plane in the vestibular area of the ts and connect the latter to the bw. Only in *Hirosella* db are ‘stacked’ as they project directly to the sphincter muscle as well. dis, diaphragmatic sphincter; o, orifice.

As a continuation of the body wall, an orthogonal grid of circular and longitudinal muscles was postulated in the ground pattern of the vestibular wall (Mukai et al., 1997; Schwaha, 2020b). However, there is considerable variation in the different phylactolaemate families (Figure 20). Stephanellids possess circular and longitudinal muscles to the same extent in their vestibular walls (Schwaha & Hirose, 2020), which also applies to fredericellids (Schwaha & Wanninger, 2012). Cristatellids and pectinatellids have pronounced longitudinal muscles and comparatively thin circular muscle fibres, whereas lophopodids and plumatellids show a reverse situation with pronounced circular muscles and delicate longitudinal muscles. *Hirosella fruticosa* lacks circular muscles in the vestibular walls and owing to its prominent longitudinal muscles, it bears a closer resemblance to Cristatellidae.

#### 4.5.2 Tentacle sheath

Usually, only longitudinal muscles were postulated in the tentacle sheath (Braem, 1890; Marcus, 1934; Mukai et al., 1997; Nitsche, 1868; Rogick, 1937). Stephanellidae, the sister taxon to all other phylactolaemates, possesses thick longitudinal muscles that are accompanied by comparatively thin circular muscles (Schwaha et al., 2016). Cristatellidae and Pectinatellidae also show both muscle sets in the tentacle sheath, but of equal thickness (Gawin et al., 2017; Schwaha, 2019). Fredericellidae also has both sets of muscles, with the circular ones being thicker (Schwaha & Wanninger, 2012). Since both, circular and longitudinal muscles in the tentacle sheath also reflect the condition of the orthogonal body wall muscles, this state is nowadays considered ancestral (Schwaha, 2020b). Nevertheless, several diverging conditions are present as well (Figure 20). The tentacle sheath includes solely longitudinal muscles in all three lophopodid genera: *Lophopus* (Marcus, 1934), *Lophopodella* (Rogick, 1937) and *Asajirella*, in which the muscle fibres bifurcate proximally (Bibermair et al., 2022; Mukai et al., 1997). Ultimately, Plumatellidae features predominately longitudinal muscles in the tentacle sheath, whereas only some circular muscle fibres are reported in the lophophoral part of the tentacle sheath of *P. fungosa* (Schwaha & Wanninger, 2012) and *Hyalinella punctata* (Gawin et al., 2017). Thus, *H. fruticosa* with continuous longitudinal and circular muscles over the entire length differs from plumatellids and is most similar to Cristatellidae and Pectinatellidae, which again shows morphological support for their closer relationship (see Saadi et al., 2022)

#### 4.5.3 Apertural musculature

Apertural muscles are important antagonists to the diaphragmatic sphincter and vestibular muscles that close the orifice and vestibulum (Schwaha, 2020b). There are two associated muscle sets: vestibular dilatators and duplicature bands. The former are thin, non‐striated muscle fibres, radially arranged around the vestibulum, while the duplicature bands comprise smooth muscle bundles with a peritoneal lining, which project from the tentacle sheath to the body wall (Mukai et al., 1997; Schwaha, 2020b). Vestibular dilatators are usually restricted with their attachment to the vestibular wall, whereas the investigated species (*H. fruticosa*, *F. sultana, I. bulgarica*) show also fibres attaching to the vestibular area of the tentacle sheath, which functionally implies that this area of the tentacle sheath is not invertible.

The duplicature bands are unique in *Hirosella fruticosa*. Typically, the duplicature bands insert at the vestibular area of the tentacle sheath and are arranged within a plane resulting in a circular arrangement or a ring of duplicature bands (Bibermair et al., 2022; Gawin et al., 2017; Schwaha, 2019; Schwaha & Wanninger, 2012). An exceptional position of lophopodids, according to which the duplicature bands insert directly at the diaphragmatic sphincter has been recently rejected (Bibermair et al., 2022). *Hirosella fruticosa* shows two alterations of this pattern: the duplicature bands do not form a distinct ring but are rather ‘stacked’ meaning they insert at different levels and not in a single plane. Moreover, they project not only to the tentacle sheath, but also to the diaphragmatic sphincter. Although the investigated fredericellids do not deviate from the ground pattern, duplicature bands of *F. sultana* occasionally appear continuous with circular body wall muscles and not, as usual, with the longitudinal ones. Previous data on fredericellid myoanatomy are scarce and were in this respect not found to differ between plumatellids and fredericellids (Schwaha & Wanninger, 2012). Unfortunately, available material was limited for this study, and future studies studying several species of fredericelllids should clarify this condition.

#### 4.5.4 Lophophoral muscles

The muscles of the lophophore and its base comprise the tentacle musculature, the musculature of the lophophoral arms and muscles of associated structures such as the epistome musculature and the muscles of the ring canal (Gawin et al., 2017; Schwaha, 2020b; Schwaha & Wanninger, 2012).

While the musculature of the lophophoral arms is prominent and includes numerous muscle bundles in species with large lophophores, for example, *A. gelatinosa, C. mucedo, P. magnifica* (Bibermair et al., 2022; Gawin et al., 2017; Schwaha, 2019), it is more delicate in plumatellids with up to five longitudinal muscle fibres, and missing in fredericellids (Gawin et al., 2017, this study) and stephanellids (Schwaha & Hirose, 2020). The present study shows delicate musculature in the lophophoral arms of *H. fruticosa* with approximately 1–3 muscle bundles, which is fewer than in other plumatellids.

In several recently investigated phylactolaemates (*H. punctata, C. mucedo, P. magnifica*), ring canal muscles in the form of radial bundles are present in the proximal lining of the ring canal (Gawin et al., 2017; Schwaha, 2019). These muscles have been overlooked in previous plumatellid studies (Schwaha & Wanninger, 2012) and are missing in lophopodids and stephanellids (Bibermair et al., 2022; Schwaha & Hirose, 2020). This study confirms their absence in fredericellids, and also in *H. fruticosa*. Consequently, ring canal musculature might have evolved twice within phylactolaemates, once in plumatellids and once in the *Cristatella*/*Pectinatella* clade or was lost in fredericellids, *Hirosella* and possibly some plumatellids.

In general, each tentacle is supplied with an abfrontal muscle on the outer side of the lophophore and a frontal tentacle muscle on the inner side (Mukai et al., 1997; Schwaha, 2020b). The abfrontal muscles are usually associated with prominent muscle bases contrary to the frontal muscles, which lack them (Bibermair et al., 2022; Gawin et al., 2017; Schwaha, 2019; Schwaha & Wanninger, 2012). The abfrontal base muscles include a pair of ascending muscle bundles, with several median muscles passing them. This fundamental architecture is the same in all Phylactolaemata, but species with larger lophophores tend to have more muscles within the lophophoral arms and tentacles (Gawin et al., 2017). However, in plumatellids and probably fredericellids, the median muscle bands show an oblique orientation between the ascending muscles (Schwaha & Wanninger, 2012). In addition, the median muscle bands are interdigitating or partly overlapping (Gawin et al., 2017). The number of median muscle bands appears smaller in Cristatellidae (Gawin et al., 2017; Schwaha, 2019). Abfrontal base muscles of the lophopodid genera *Asajirella* and *Lophopodella* consistently have five median muscle bands, possibly indicating a certain taxon specificity (Bibermair et al., 2022). Stephanellids differ and lack median muscles over the longitudinal ones. Instead, several rather thick muscle bundles are arranged in a zig‐zag at the abfrontal base (Schwaha & Hirose, 2020). *Hirosella fruticosa* shows another variation of abfrontal base muscles and has only some small packages of oblique muscle bands. Distal of the latter a short gap is present and followed by two muscle bundles that join and ascend as tentacle muscle. Consequently, *H. fruticosa* shows different and much smaller abfrontal base muscles than *Pectinatella* or *Cristatella*. In *F. sultana* they are similar to *H. fruticosa* and are less prominent than in other families (Bibermair et al., 2022; Gawin et al., 2017; Schwaha & Wanninger, 2012; Schwaha, 2019). Some muscle bands are present at the proximal part of the abfrontal base muscle, but not as separated from the ascending tentacle muscle bundle as in *H. fruticosa*. However, this notion supports a previous analysis according to which there is a gap between the proximal muscle bands and the ascending abfrontal tentacle muscle in *F. sultana* (Schwaha & Wanninger, 2012). In general, the abfrontal base muscles of *F. sultana* and *H. fruticosa* are more similar to each other than to plumatellid abfrontal bases. Sometimes the oral abfrontal base muscles are connected to the pharyngeal musculature in *F. sultana*, which has not been described for fredericellids before. Taxa with large lophophores have their abfrontal base muscles connected to the musculature of the lophophoral arms, e.g., *Asajirella*, *Lophopodella*, *Cristatella* and *Pectinatella*. Oral tentacles of these groups are embedded in the pharyngeal epithelium but are not necessarily connected to it (Bibermair et al., 2022; Gawin et al., 2017; Schwaha & Wanninger, 2012).

In contrast to the abfrontal muscle bases, the frontal muscle bases of all phylactolaemates except *Stephanella* (Schwaha & Hirose, 2020) have proximal rootlets (Bibermair et al., 2022; Gawin et al., 2017; Schwaha, 2019, 2020a; Schwaha & Wanninger, 2012). These rootlets differ in number depending on their position on the lophophore and also the species. In *Pectinatella* and *Cristatella*, the oral tentacles possess two to three rootlets, which in *Cristatella* are also sometimes laterally connected (Gawin et al., 2017; Schwaha, 2019). The presence of two to three rootlets in *Hirosella fruticosa* is another morphological character that supports the hypothesis of a closer relationship between *Hirosella*, *Cristatella* and *Pectinatella* (Saadi et al., 2022). Three rootlets appear to be restricted to the lateral tentacles of *H. fruticosa*, whereas the frontal tentacle muscles of the oral‐most tentacles show only two rootlets. Also, the oral tentacles emerge from a circular muscle ring around the pharynx in *Cristatella* and *Pectinatella* and not directly from the pharynx musculature (Gawin et al., 2017), which is reminiscent of the situation observed in *H. fruticosa*, but is also present in plumatellids (Schwaha & Wanninger, 2012). The lack of this basal interconnecting ring in fredericellids indicates that it was most likely lost in this family and evolved at the branch separating Plumatellidae from all remaining families except Lophopodidae and Stephanellidae. The frontal tentacle muscles of the lophophoral arms are connected to the muscles of the lophophoral arms via one or two rootlets in *H. fruticosa*. In tentacles closer to the lophophoral concavity, a second rootlet seems to be missing or not detectable, which was also observed in *Cristatella* and *Pectinatella* (Gawin et al., 2017). In contrast, frontal tentacles muscles on the lophophoral arms of plumatellids have just one rootlet (Gawin et al., 2017; Schwaha & Wanninger, 2012). Ultimately, fredericellids differ in their frontal tentacle muscles from other families. First, their bases are conspicuously large in the lophophoral concavity and consist of several parallel muscle fibres. Second, they are all interconnected to adjoining tentacles, regardless of their position on the lophophore. Such lateral connections are also common in *H. fruticosa* and can occur in *Cristatella* (Gawin et al., 2017; this study). In contrast, such connections are missing in plumatellids, lophopodids and presumably also pectinatellids and stephanellids (Bibermair et al., 2022; Gawin et al., 2017; Schwaha & Hirose, 2020; Schwaha & Wanninger, 2012). The function of such lateral interconnections remains unknown.

In summary, tentacle musculature of *H. fruticosa* distinctly differs from plumatellids, since they lack multiple frontal base rootlets and lateral connections. Instead, the abfrontal muscles show similarities to fredericellids, and the frontal base muscles to Cristatellidae. Considerable variation of the finer anatomy of the lophophore is present in phylactolaemates. Thus, an ultrastructural examination of the lophophoral base over the entire range of families would be desirable in the future.

#### 4.5.5 Epistome musculature

The last set of muscles at the lophophoral base is the epistome musculature. Three different conditions have been described in phylactolaemates (Bibermair et al., 2022; Gawin et al., 2017; Schwaha, 2020a): (1) muscle fibres are embedded into the peritoneal lining of the epistome and form a muscular basket, (2) individual muscle bundles traverse the epistomial coelom (Schwaha, 2020a, 2020b) or (3) both types at once. The early‐branching stephanellids as well as pectinatellids possess traversing muscles in the epistome (Bibermair et al., 2022; Gawin et al., 2017; Schwaha, 2018; Schwaha & Hirose, 2020). Most plumatellids, fredericellids and cristatellids possess the muscular basket (Gawin et al., 2017; Schwaha & Wanninger, 2012; Schwaha, 2019). The plumatellid *Hyalinella punctata* and lophopodids have both sets of muscles (Bibermair et al., 2021; Gawin et al., 2017). Our study confirms traversal muscles for the epistome of *H. fruticosa*, which are absent in Plumatellidae (Schwaha & Wanninger, 2012). At the same time, confocal scans of *Hirosella* look rather similar to *Fredericella*, which has the epistomial muscular basket (Schwaha & Wanninger, 2012). The true nature of the epistomial muscles can be difficult to distinguish on sections when structures have collapsed or shrunken, or in confocal scans when fibres are too delicate to be properly identified. Nevertheless, some confocal scans of *F. sultana* show thin fibres in the centre of the epistome as well, which indicates that lophopodids, the plumatellid *H. punctata*, the fredericellid *F. sultana* and *Hirosella* might have both sets of epistome muscles. Ultrastructural data of the epistome are limited and focused on its coelomic cavity. Lining myoepthelial cells were reported (Gruhl et al., 2009), but further ultrastructural investigations are required to clarify the nature of the different epistomial muscle types, especially in smaller species such as fredericellids.

## 5 CONCLUSION

The present study provides the first modern morphological analysis of the species formerly addressed as *P. fruticosa* and includes the first morphological analysis of *Internectella bulgarica*, the sole species of the second fredericellid genus. Our data on colony morphology and zooidal characters support the hypothesis that *P. fruticosa* does not belong to Plumatellidae and warrants the creation of the new genus, *Hirosella* gen. nov. including its separate family. Some main characteristics of the genus are the highly erect colonies, proportions of the gut and lophophore which are fredericellid like, whereas the lophophore itself is horseshoe‐shaped. The attachment of the duplicature bands in *Hirsosella* is distinctive, with varying insertion levels into the tentacle sheath and the diaphragmatic sphincter. The lack of circular muscles in the vestibular wall and the continuous orthogonal tentacle sheath muscles distinguish *H. fruticosa* further from plumatellids, but also show similarities to Cristatellidae and Pectinatellidae. The intertentacular membrane attachment of *Hirosella* is similar to *Cristatella*. In general, our data show morphological support for a clade of *Hirosella*, *Cristatella* and *Pectinatella*, but also their relatedness to fredericellids. The limited data we could provide on *Internectella bulgarica* shows clear differences to other fredericellids, but future molecular and morphological work is required to assess its phylogenetic position.

## AUTHOR CONTRIBUTIONS

Julian Bibermair and Thomas Schwaha collected the material. Julian Bibermair conducted all lab work, analysed the data, prepared all figures and drafted the manuscript. Thomas Schwaha analysed the data, improved the manuscript and designed and supervised the study.

## CONFLICT OF INTEREST STATEMENT

The authors declare no conflict of interest.

## Data Availability

The data that support the findings of this study are available on request from the corresponding author. The data are not publicly available due to privacy or ethical restrictions.
